# Supplementary material for: How PrEP delivery was integrated into public ART clinics in central Uganda: A qualitative analysis of implementation processes
Source: PLOS Glob Public Health. 2024 Mar 7;4(3):e0002916. doi: 10.1371/journal.pgph.0002916 (PMC10919847; doi:10.1371/journal.pgph.0002916)
Supplement: S1 File — The qualitative study was part of behavioral research collected in the stepped-wedge cluster randomized trial. (PDF) [file pgph.0002916.s002.pdf]

PROTOCOL

Integrated PrEP and ART delivered in Ugandan public health clinics to improve HIV and ART outcomes for HIV serodiscordant couples

The Partners PrEP Program

Version 3.0

28 May 2020

Funding:

U.S. National Institute for Mental Health (R01MH110296)

Contents

STUDY TEAM ... 4
I. SUMMARY ... 5
II. BACKGROUND ... 6
III. STUDY METHODS ... 11
Overall Design ... 11
Aims ... 11
Population ... 12
Location ... 12
Eligibility ... 12
Sample size and study power ... 13
Recruitment ... 13
Clinic randomization ... 13
Launch of intervention delivery ... 14
Study procedures ... 14
Screening and enrolment ... 14
Follow up ... 15
Procedural tables ... 15
Retention and exit from research activities ... 17
Special circumstances ... 17
Safety Monitoring ... 17
Medications ... 17
Qualitative interviews and field observations ... 18
Microcosting ... 19
Intervention fidelity ... 19
IV. DATA COLLECTION ... 19
V. DATA ANALYSIS ... 20
Analysis of effectiveness of the integrated PrEP and ART intervention ... 20
PrEP as modeled behavior for ART use ... 20
Analysis of barriers and facilitators of PrEP and ART use ... 20
Analysis of qualitative interviews and field observations ... 20
Estimating cost-effectiveness and budget impact ... 20
VI. HUMAN SUBJECTS CONSIDERATIONS ... 21
Study oversight ... 21
Informed Consent ... 21
Risks ... 21
Benefits ... 22

Care for persons identified as HIV positive 22
Treatment for injury 22
VII. CLINICAL RESEARCH SITE 23
VIII. REFERENCES 24

STUDY TEAM

Makerere University, Kampala, Uganda

Elly Katabira, FRCP
Andrew Mujugira, MBChB, PhD
Timothy Muwonge, MBChB, MPH

University of Washington, Seattle, USA

Renee Heffron, PhD, MPH
Jared Baeten, MD, PhD
Deborah Donnell, PhD
Ruanne Barnabas, MBChB, DPhil
Jane Simoni, PhD
Caitlin Scoville, MPH
Katherine Thomas, MS

Harvard University, Boston, USA

Norma Ware, PhD
Monique Wyatt, BA

I. SUMMARY

There is great progress towards controlling the HIV epidemic and tools that could end HIV transmission are now known. Antiretrovirals are profoundly important biomedical tools including antiretroviral therapy (ART) used by HIV positive individuals and pre-exposure prophylaxis (PrEP) used by HIV negative individuals. In 2015, the World Health Organization issued a recommendation of ART initiation for all individuals immediately following an HIV diagnosis and PrEP use by HIV negative individuals with substantial HIV risk. Despite the tremendous benefits of ART and growing widespread availability, delays in ART initiation and difficulties establishing high adherence are common. Delays in ART use are especially concerning for HIV-positive individuals who are in established sexual relationships with HIV-negative partners–i.e. serodiscordant couples–as negative partners face an extremely high risk of infection prior to HIV viral suppression by positive partners. For individuals lacking HIV protection from a partner's ART use, PrEP offers an immediate prevention solution.

For more than 10 years, we have been studying the use of PrEP and ART for HIV prevention among HIV serodiscordant couples in Uganda, through placebo-controlled proof-of-concept randomized trials and implementation science-driven delivery studies. In early 2015, we demonstrated that an integrated PrEP and ART strategy (with PrEP used until ≥6 months of ART use by the HIV-positive partner) nearly eliminates HIV transmission within high risk HIV serodiscordant couples. To date, this work has been conducted in research clinics permitting extended time with study participants to provide detailed information about these novel prevention strategies and resources to promote high retention and adherence. It is not clear that our integrated PrEP and ART strategy would be delivered as effectively in a public health setting by staff without in-depth prescribing experience.

Data from this study also suggest - but were not designed to confirm - an effect of the integrated PrEP and ART intervention on ART outcomes with ART initiation rates ~90% within 12 months of first referral and with >90% of HIV-positive partners virally suppressed within 6 months of initiation. This hypothesis merits further study, as an added value of PrEP use to improve ART initiation and adherence would provide further reason to advance PrEP delivery for HIV serodiscordant couples. Additional unaddressed questions include how the use of PrEP and ART interact within couples and the cost of adding PrEP to already existing services.

Through close collaboration with the Ugandan Ministry of Health, we will conduct a stepped wedge cluster randomized trial of integrated PrEP and ART for HIV serodiscordant couples in public health clinics to study the effectiveness of delivering integrated PrEP and ART at public health clinics on PrEP initiation and adherence, ART initiation and adherence. Additional behavioral components and qualitative research will be incorporated to determine the degree to which PrEP-taking is a modeled behavior and to characterize barriers and facilitators to the use of ART and PrEP and the processes by which PrEP and ART use interact within couples. Programmatic costing analyses will be conducted to estimate programmatic costs and guide intervention scale up.

II. BACKGROUND

Antiretrovirals for HIV prevention

The past thirty years of HIV research and public health programming have resulted in great progress towards controlling the HIV epidemic and reducing the number of new infections occurring each year. The World Health Organization (WHO) has recently stated that the tools to end AIDS are now available.[1] Antiretrovirals – used by HIV-positive individuals to treat HIV infection (ART) and by HIV-negative individuals prophylactically to prevent HIV infection (PrEP) – are central to discussions about ending the AIDS epidemic. However, much work remains to optimize the delivery of antiretrovirals to millions of people with and at high risk of HIV using strategies that will achieve the greatest effectiveness and coverage possible.[2] Uganda carries the 5th highest HIV burden in the world, with 1.6 million people living with HIV, and 140,000 new infections and 63,000 AIDS-related deaths in 2013.[1] To achieve significant impact in reducing these trends among Ugandans, effective and scalable delivery systems and high uptake of antiretroviral-based interventions are critical.

HIV prevention among HIV serodiscordant couples

The source of up to half of all new HIV infections in Africa has been estimated to be a stable heterosexual partner, making HIV serodiscordant couples – in which one partner is HIV-positive and the other is HIV-negative – a priority target population for HIV prevention interventions.[3, 4] Following a diagnosis of HIV serodiscordance, couples face difficult challenges identifying ways to preserve their relationship and keep the HIV-negative partner uninfected amidst desires for intimate sex, pregnancy, and general normalcy in their lives.[5, 6] Antiretrovirals offer solutions for the dilemmas faced by HIV serodiscordant couples. Their use as PrEP by the HIV-negative partner and as ART for the HIV-positive partner can be integrated to provide immediate protection as well as a longterm solution.[7, 8]

For HIV serodiscordant couples, PrEP provides immediate protection to an HIV-negative partner prior to an HIV-positive partner's ART initiation and viral suppression, forming the basis of an integrated PrEP and ART strategy. Multiple randomized clinical trials among a variety of populations with HIV risk have demonstrated the efficacy of PrEP, administered as a once daily tenofovir-based regimen.[9-12] 2015 WHO guidelines on antiretrovirals recommend PrEP for people with substantial HIV risk.[13] The release of this guidelines was met with great enthusiasm for advancing PrEP implementation in resource-limited settings, with rapid approvals by drug authorities in Kenya and South Africa and the development of implementation plans.[14] Amidst great enthusiasm for PrEP and optimizing its delivery in many resource-limited settings, the impetus for scientific research now is to model approaches to PrEP delivery that target high-risk populations, such as HIV serodiscordant couples, to understand whether the integrity of intervention can be maintained in public health clinics and the impact of the intervention on HIV outcomes.

ART use with high adherence and maintenance of viral suppression is the ultimate goal for long-term prevention of HIV transmission within HIV-serodiscordant couples and optimal health for HIV-positive individuals.[15, 16] However, the period between ART initiation and viral suppression (up to 6 months after ART initiation) remains a period of HIV risk and delays in ART initiation are common and extend the period of HIV risk for HIV-negative partners.[17, 18] Worldwide, 20-50% of HIV-positive individuals are estimated to be lost from the health system between HIV diagnosis and ART initiation.[19] In a recent systematic review of studies conducted in sub-Saharan Africa, an average of 68% of individuals eligible to initiate ART actually did so within the study observation periods (a range of 3 months to >3 years).[20] Social and individual barriers to ART initiation include a state of "feeling healthy" that

dissuades individuals from taking medications, the prevailing opinion that ART initiation signals death, stigma and fear of disclosure to partners, family or friends, fears of Identifying drug toxicity, financial constraints impeding clinic visits and delays receiving laboratory results.[19, 21-24] Thus, research that identifies methods to improve ART initiation within the context of the local environment is high priority.

Figure 1. An integrated PrEP and ART strategy for HIV serodiscordant couples with PrEP used by the HIV-negative partner until ART use by the HIV-positive partner provides HIV protection

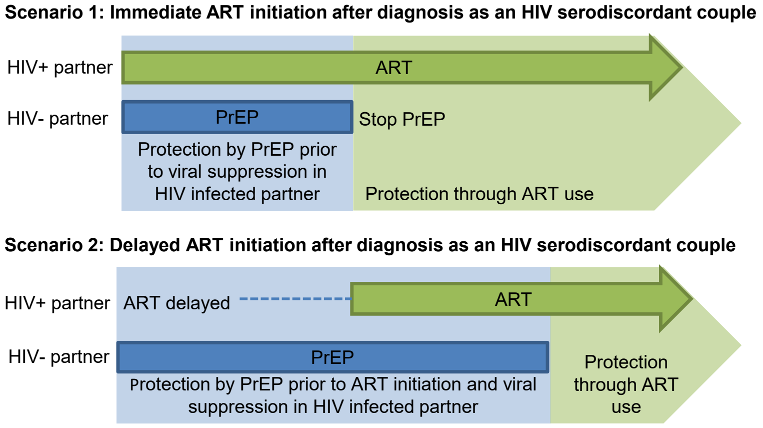

Integrated PrEP and ART for HIV serodiscordant couples – the Partners Demonstration Project

We have recently concluded testing the impact of an integrated PrEP and ART strategy in our ongoing Partners Demonstration Project among 1,013 HIV serodiscordant couples at 4 clinical research sites in Uganda and Kenya. In this model, PrEP provides protection against HIV acquisition prior to viral suppression in the HIV-positive partner (Figure 1, Scenario 1). For couples in which the positive partner does not initiate ART immediately upon diagnosis, the period of HIV risk to the negative partner is extended and PrEP is used for a longer period of time (Figure 1, Scenario 2).

PrEP use could impact ART use

Results from the Partners Demonstration Project showed that the integrated PrEP and ART strategy nearly eliminates HIV transmission between couples (Figure 2) and were pertinent for WHO guidelines recommending PrEP use by individual with substantial risk.[13, 25] Additional preliminary data suggest that the integrated PrEP and ART strategy may accelerate rates of rates of ART initiation and viral suppression among HIV-positive partners (Figure 3). Nearly 90% of all HIV-positive individuals initiated ART within 6 months of receiving a referral (Figure 3, panel A; Mujugira et al., in preparation). By comparison, in the Partners PrEP Study, our prior double-blind, randomized, placebo-controlled clinical trial of PrEP for HIV prevention among 4,747 HIV serodiscordant couples in 9 sites in Kenya and Uganda, 50% of HIV-positive participants had not initiated ART within 6 months and 30% had not initiated within 12 months of their first referral (Figure 3, panel B).[18] This hypothesis merits further study, as an added value of PrEP use to improve ART initiation and adherence would provide further reason to advance PrEP delivery for HIV serodiscordant couples.

Figure 2. Observed versus expected HIV infections in the Partners Demonstration Project. The integrated PrEP and ART strategy nearly eliminated HIV transmission risk. (25)

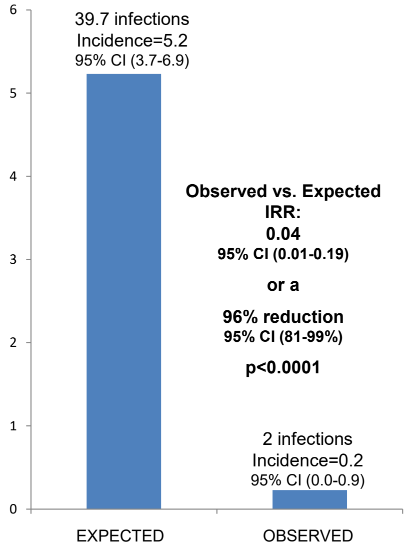

Based on Social Learning Theory (or social cognitive theory), we hypothesize that PrEP use by an HIV-negative partner within an HIV serodiscordant couple provides an opportunity for antiretroviral use to be modeled by HIV-negative partners using PrEP and this may be driving the reduction in ART delays that we have observed in our study.[26] Through the opportunity to witness an HIV-negative partner managing medication side effects, daily pill burden, and HIV-risk related stigma, HIV-positive partners may develop greater self-efficacy for their own medication taking and the perception that the attainment of optimal health is worthwhile for themselves, their partner, and their relationship.[27] Interventions capitalizing on social learning theory have been implemented using role models to demonstrate ART use and peer supporters to influence multiple HIV-related health domains.[28] Provider guidance and structured couples-based counseling have been noted as important components to integrate into interventions that leverage partners to improve ART outcomes.[29, 30] These

Figure 3. Cumulative probability of initiation ART by CD4 count after ART referral among HIV-positive members of HIV serodiscordant couples in A) the Partners Demonstration Project open-label PrEP and ART delivery study (25) and B) Partners PrEP Study randomized clinical trial of PrEP (9)

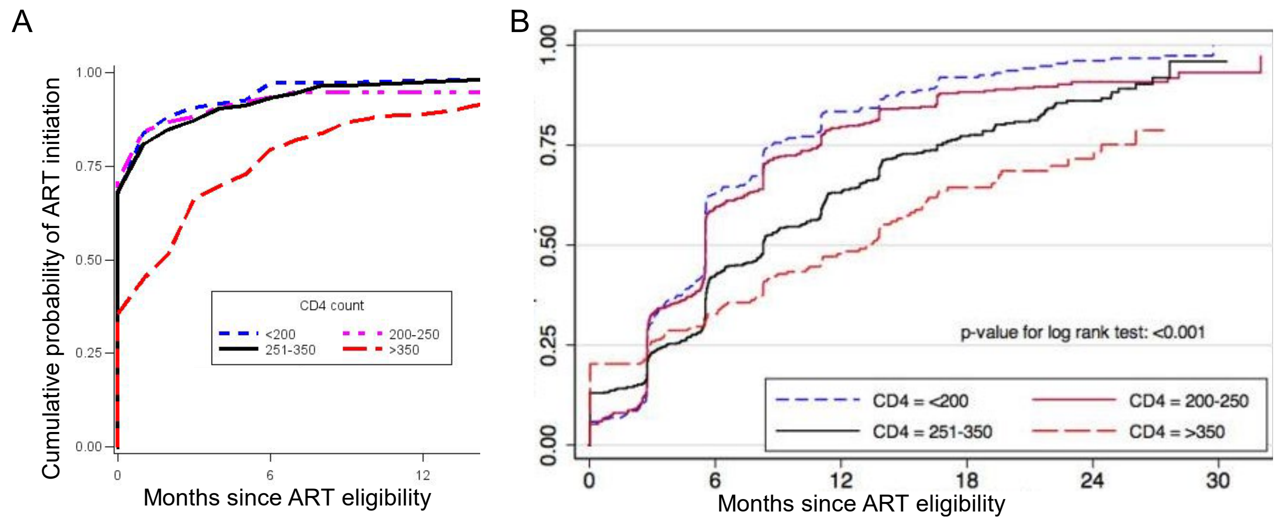

interventions also foster communication between HIV-positive individuals and their support network of providers and partners and have the potential to influence long-term ART outcomes. By this mechanism, an integrated PrEP and ART strategy that actively engages both couples to use antiretrovirals and support one another's adherence may improve rates of ART adherence and viral suppression in the HIV-positive partner.

Table 1. Components of HIV prevention delivery for HIV serodiscordant couples at public health clinics following the standard of care and components added when the integrated PrEP and ART intervention is implemented.

HIV prevention for serodiscordant couples with standard of care	Additional components with implementation of integrated PrEP and ART
• Behavioral counseling<br>• Condom provision<br>• ART counseling & provision for HIV-positive partner<br>• 6-monthly CD4 testing<br>• HIV RNA testing if virologic failure is suspected	• Counseling on PrEP, adherence to PrEP<br>• Counseling on integrated PrEP and ART<br>• Provision of PrEP<br>• Assessment of acute HIV infection in HIV-negative partner<br>• 6-monthly renal function monitoring of HIV-negative partner<br>• 6-monthly HIV RNA testing for HIV-positive partner (research procedure)<br>• Blood samples archived for tenofovir testing and additional tests in the event of seroconversion (research procedure)

Components of the integrated PrEP and ART intervention

Public health clinics in Uganda already provide HIV serodiscordant couples with basic behavioral counseling, condoms, and ART counseling, provision, and accompanying lab tests (Table 1). Based on our procedures followed in the Partners Demonstration Project, the integrated PrEP and

ART intervention adds clinical components for PrEP delivery including counseling on PrEP and adherence, behavioral counseling on how PrEP and ART can be integrated, PrEP medication with a 3-monthly supply and HIV testing and acute HIV assessment prior to refills, and 6-monthly creatinine testing. These procedures are all in accordance with WHO clinical guidelines. Additional research components to enhance understanding of how couples make choices about PrEP and ART use include: 6-monthly HIV RNA testing for the HIV-positive partner, archival of blood samples, tenofovir testing for HIV-negative partners, and behavioral assessments.

Counseling to accompany the delivery of integrated PrEP and ART

Behavioral counseling is an essential component of the integrated PrEP and ART intervention. Based on our work in the Partners Demonstration Project, we have identified topics and topical flow for serodiscordant couples HIV counseling (Figure 6) as well as counseling messages specific to PrEP and ART (Figure 7, manuscript in preparation). We will use these messages to form the foundation of HIV prevention counseling for couples. These messages were developed at the outset of Partners Demonstration Project based on scientific literature and our experience conducting the Partners PrEP Study, including the open-label extension period that followed the discontinuation of the placebo arm.[9, 31] After initiation of the Partners Demonstration Project, an iterative approach was used to identify the progression of counseling and topics related to ART, PrEP and their integration as it was naturally occurring at study clinics. Study staff involved in counseling – counselors, clinicians and pharmacists – from each site participated in group discussions and one-on-one discussions with each other and study investigators to identify counseling topics and the messages most commonly used and of greatest priority. These messages were refined to ensure the use of the comprehensible and accurate language. This process also identified messages that caused participant confusion, common questions, and analogies used to frame the messages to increase participant comprehension. Prior to finalizing the messages, the entire study team reviewed the package of messages and suggested final revisions.

The general counseling approach is didactic and includes components to convey key information to couples with active, supportive counseling to address concerns and barriers. Providers initiate discussion of key concepts and themes as couples progress through phases

Figure 6. Topical flow for counseling HIV serodiscordant couples, including discussions about integrated PrEP and ART (manuscript in preparation)

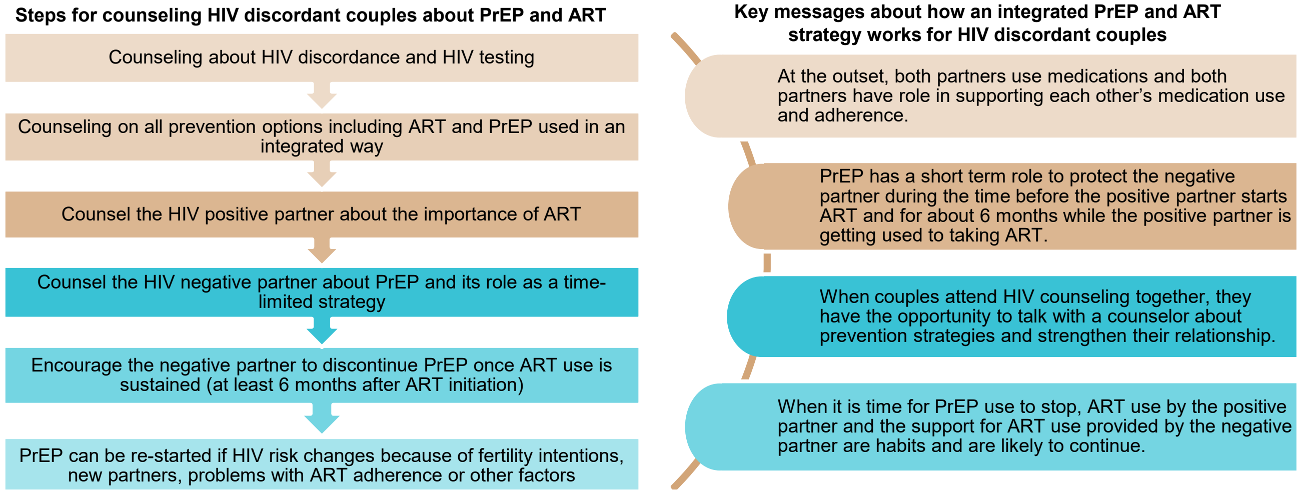

Figure 7. Key messages about ART use (in blue) and PrEP use (in brown) refined through iterative discussions with counselors and clinicians delivering integrated PrEP and ART to HIV serodiscordant couples in the Partners Demonstration Project (Morton et al. in press).

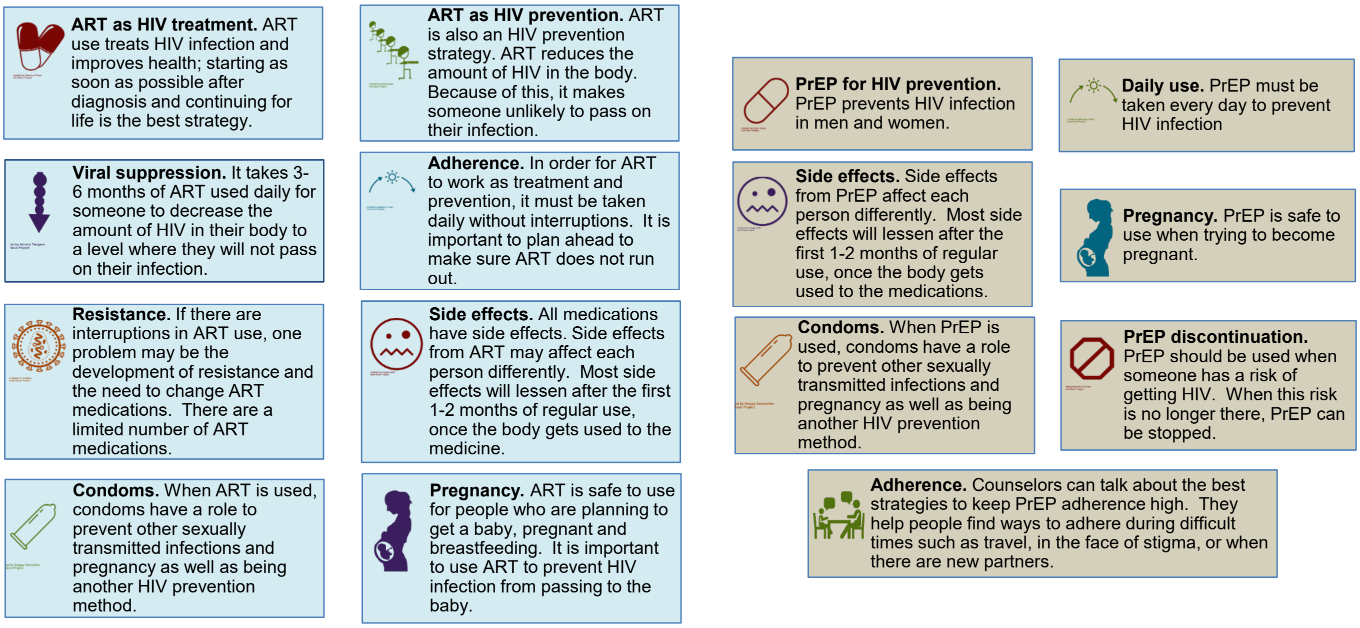

of learning and accepting their serodiscordant status, recognizing ongoing risk, and resolving to remain a couple despite HIV transmission risk.[32]Counseling sessions occur every 3 months for approximately 30-60 minutes with ad hoc sessions provided upon request from the couple. This frequency and length of counseling sessions was successful in the Partners Demonstration Project and is a manageable length of time to add to already overburdened health care providers.

Providers initiate discussion about HIV serodiscordance and the reality of this situation, the efficacy of PrEP and ART for prevention, willingness to initiate PrEP and ART, strategies to maintain daily adherence to PrEP and ART, the time-limited nature of PrEP use and risk situations that merit longer term use, other HIV risk reduction strategies (condom use, medical male circumcision, and treatment of sexually transmitted infections), and couples-based communication. It is common for providers to initiate discussion about fertility desires, HIV risk reduction during pregnancy attempts, and optimization of pre-pregnancy health since pregnancy and family building are common goals of Ugandan couples. The technical training team has extensive experience with all of these topics and our intervention manual will provide detailed examples of how to initiate these discussions and appropriate safer conception strategies to recommend.

COVID-19 impact

The COVID-19 pandemic entered Uganda in March 2020. The public health response in Uganda included safety protocols such as shelter-in-place and transport restrictions, shortly after the first cases were confirmed. The impact of COVID-19 has disproportionately affected vulnerable populations who are more at risk for food insecurity, gender-based violence, and financial insecurity. For HIV serodiscordant couples, COVID-19 may lead to reduced access to PrEP and ART and providers may be inaccessible due to social distancing requirements or the healthcare system being overwhelmed with COVID patients. This study will also provide the opportunity to gather data on provider and participant experiences with PrEP and ART access during COVID-19 and the impact of COVID-19 on behavior and HIV risk.

Overall Impact

The proposed work will build upon our work in the area of PrEP efficacy and delivery but it is designed and powered to give specific attention to the delivery of integrated PrEP and ART in Ugandan public health clinics using a sustainable and scalable strategy. Results from this study will provide compelling data to inform widespread delivery of integrated PrEP and the integration of PrEP and ART for HIV serodiscordant couples as a component of Ugandan national HIV prevention policy during and external to the time of COVID-19.

III. STUDY METHODS

Overall Design

We will launch a stepped-wedge cluster randomized trial in 12 public health clinics in Kampala, Uganda encompassing 3 total steps with 4 clinics initiated at each step (Figure 5). Each step of the trial will be initiated with 1) clinic-wide training (on HIV prevention for serodiscordant couples, the prevention benefits of PrEP and ART, and integrated delivery of PrEP and ART for serodiscordant couples) and 2) provision of PrEP stocks at each of the clinics. Throughout the study period, clinics delivering the intervention will have regular visits from members of the training team to walk through the delivery at their clinic and identify components of the intervention that can be made more efficient and higher quality. Each site will continuously enroll HIV serodiscordant couples into a cohort (of approximately 30 per clinic per step) for the measurement of outcomes.

Figure 5. Schematic of stepped wedge study design. Outcome assessment (shown in purple) begins at baseline. A new group of clinics initiates the intervention at the beginning of each 9-month step (shown in blue) and provides integrated PrEP and ART through the duration of the study (shown in green).

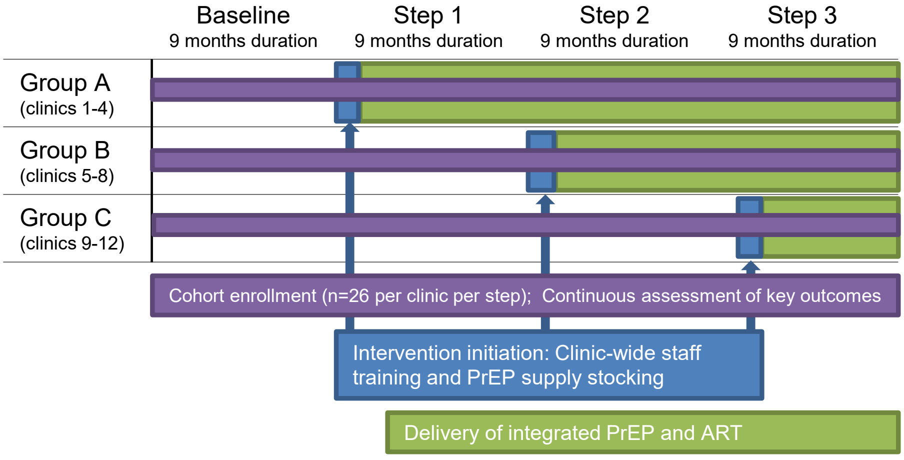

Aims

1. **To determine the effectiveness of delivering the integrated PrEP and ART intervention <u>at public health clinics</u> in Kampala, Uganda, on: a) PrEP initiation, b) PrEP adherence, c) ART initiation, and d) ART adherence among HIV serodiscordant couples.**

   We will conduct a stepped-wedge cluster randomized trial, incorporating a staged introduction of the PrEP and ART integrated intervention, to initiate delivery of PrEP at 12 public health clinics in Kampala, Uganda. HIV serodiscordant couples at each clinic will be engaged to join a cohort that is followed with research procedures to capture outcome data.

   Outcomes: Behavioral and biological markers of PrEP and ART uptake and adherence. These outcomes will be compared between clinics that have begun implementing the intervention versus clinics randomized to launch implementation later in the study.

2. **To determine the degree to which PrEP-taking is a modeled behavior and its contribution to the potential effect of the integrated PrEP and ART intervention on ART initiation and adherence.**

   We will measure the degree to which PrEP-taking behavior is modeled by HIV-negative partners and the degree to which it is witnessed by HIV-positive partners.

   Outcomes: Quantitative assessments of modeled behavior related to PrEP use. Statistical models will estimate the degree to which modeled behavior accounts for any association between the intervention and ART initiation and adherence.

3. **Through quantitative and qualitative research with HIV serodiscordant couples and HIV providers, we will characterize: a) potential barriers and facilitators to the use of ART and PrEP, including the gender of the HIV positive partner, relationship dynamics, and intimate partner violence, b) the processes by which PrEP and ART use interact within couples, and c) perspectives about the COVID-19 pandemic on ART and PrEP.**

   We will use validated scales of medication self-efficacy, relationship power, alcohol use, and intimate partner violence to assess the role of each of these factors as barriers and facilitators to PrEP and ART use. Through repeated in depth interviews and observations of counseling sessions, we will characterize the meaning of the intervention for couples and describe implementation challenges that occur during and external to the time of COVID-19.

4. **To estimate the programmatic costs of providing integrated PrEP and ART to HIV serodiscordant couples.**

   We will conduct micro-costing, cost-effectiveness, and budget impact analyses, including estimating opportunity costs for people who elect to not use PrEP, to guide approaches for intervention scale up.

Population

1. HIV serodiscordant couples from the Kampala, Uganda area
2. HIV care and treatment providers from Kampala public health clinics

Location

The study will initially be implemented in the Kampala City Council (KCC) clinics and Wakiso district facilities, across multiple implementing partners to ensure that the systems are robust to different operating structures. The Infectious Diseases Institute has in the past supported capacity building by exporting their best practice model of HIV care delivery to the health service providers working with KCC. When appropriate, the delivery of PrEP will be modeled around the delivery of ART and partner testing will be encouraged in order to identify HIV discordant couples. We shall support health units that are struggling with retention of patients on ART and patients with ART adherence challenges.

Eligibility

<u>For both members of the couple</u>

- Age ≥18 years
- Able and willing to provide informed consent

- Sexually active with each other
- Willing to engage with the clinic system as a couple

For HIV-positive members of the couples (index participants)
- HIV-positive, according to national HIV testing algorithm
- Recent diagnosis as a member in an HIV serodiscordant couple
- Not currently enrolled in an HIV treatment clinical trial

For HIV-negative members of the couples (partner participants)
- HIV-negative, according to national HIV testing algorithm
- Recent diagnosis as a member in an HIV serodiscordant couple
- Not currently enrolled in an HIV treatment clinical trial
- Not currently using PrEP
- Eligible for PrEP, according to WHO or Ugandan national guidelines

HIV care and treatment providers for qualitative interviews
- Age ≥18 years
- Able and willing to provide informed consent
- Providing HIV care, treatment and/or prevention services at health clinics in the Kampala, Uganda area

Sample size and study power

This study will include approximately 1440 HIV serodiscordant couples in a longitudinal cohort with quantitative and qualitative data collection and approximately 36 HIV care and treatment providers for qualitative data collection.

Our sample size for the cohort of HIV serodiscordant couples was chosen to detect a change in ART initiation rates at 3 months; based on historical rates of ART initiation in Ugandan public health clinics, we expect 50% of participants to initiate ART at 3 months, and have determined that we will have 80% power to detect changes in the initiation of ART from 50% to 65% (or higher) with 23 couples per step, 4 steps including the baseline step, and 4 clinics initiating the intervention per step (12 total clinics). Our sample size of approximately 30 couples per step per clinic (1440 total couples) incorporates an expected 10-15% loss to follow up and is robust to the assumed level of correlation within versus between clinics.

Recruitment

Each clinic participating in the proposed study will deploy methods most appropriate for their patient population and setting to recruit HIV serodiscordant couples to the clinic for HIV testing and to receive the intervention or standard ART care, depending on whether the clinic has initiated the intervention yet. Strategies include collaborating with existing HIV counseling and testing centers to become a referral site for newly diagnosed HIV serodiscordant couples and conducting outreach activities to community markets and other public gathering places to encourage couples-based HIV testing at the clinic. Clinics will receive training on best practices for recruiting serodiscordant couples and be provided with key talking points (provided in an Appendix).

Clinic randomization

Clinics will be randomly assigned to start intervention delivery at one of the three study steps, 4 clinics per step; randomization of clinics to the order of intervention steps will be

stratified by size and region and will take place at a public meeting, led by the study statistician including the clinics and their communities.

Launch of intervention delivery

The initiation of intervention delivery will involve 2 key features: 1) clinic-wide training on delivering integrated PrEP and ART and 2) provision of the first stocks of PrEP to each clinic. Once PrEP stocks are in place and clinic staff are adequately trained on all components of delivering integrated PrEP and ART, all individuals being seen at the clinic for HIV testing will be encouraged to return with a partner for couples-based HIV testing. Identified HIV serodiscordant couples will be encouraged to have the HIV-positive partner initiate ART and to have the HIV-negative partner initiate PrEP.

Clinic-wide training. At the launch of each step, staff from the 4 clinics launching delivery of integrated PrEP and ART during the step will be trained on HIV prevention counseling for serodiscordant couples, prevention benefits of PrEP and ART, specifics of delivering of integrated PrEP and ART, and good clinical research practices. Starting with the materials developed to deliver integrated PrEP and ART and train study staff in the Partners Demonstration Project (Figures 6 and 7), we will develop a comprehensive set of training manuals and materials that are specific to the Ugandan context and could be used during the scale up of PrEP delivery to other Ugandan clinics. The final component of clinic wide training will include a one-day onsite visit by the training team to each clinic to observe mock patient visits and counseling sessions and discuss patient flow efficiencies with the clinic team. At the end of this visit, the training team will complete a proficiency checklist. Subsequently, the training team will work closely with the clinic on a daily basis so to ensure the clinic achieves proficiency in each delivery element before being activated to deliver the intervention.

PrEP stocking. Following the training, each clinic will receive a PrEP supply and initiate delivery of integrated PrEP and ART.

Study procedures

Specific procedures for 1) the implementation and 2) research procedures are described in subsequent tables and brief descriptions are provided below. At any time, a clinic will be implementing either the standard of care for HIV serodiscordant couples or the integrated PrEP and ART intervention. Implementation procedures under the standard of care are according to national Ugandan ART clinic guidelines and national PrEP guidelines. When clinics initiate delivery of integrated PrEP and ART, additional procedures are added and these are described in tables below. Research procedures are the same for both prevention strategies.

Screening and enrolment

HIV serodiscordant couples engaged by the public health clinics will undergo minimal research procedures prior to study enrollment including informed consent, collection of demographic data, and renal function screening for the HIV-negative partner. Couples in which the HIV-negative partner has abnormal renal function will not be eligible for the integrated PrEP and ART intervention, and thus not eligible to be in the study cohort but these couples will receive intensive counseling on the importance of ART initiation and its benefits for HIV prevention and treatment. As much as possible, we will use point-of-care diagnostics to reduce the time between screening and enrollment. Information required to determine eligibility for research procedures is routinely collected as part of the intervention. We will work with participating clinics to determine eligible couples from the inclusion criteria that can be ascertained from the medical charts at the clinic. Couples who meet the study's inclusion criteria will be invited

for eligibility confirmation and to ascertain their willingness to participate in research procedures. At enrollment, all eligible couples will receive the HIV prevention interventions offered by the clinic (integrated PrEP and ART for clinics that have initiated the intervention, standard of care for the clinics that are yet to initiate the intervention).

Follow up

Couples will be scheduled for visits at the clinic on a quarterly basis, which is in line with the current schedule for ART patients. As part of routine public health clinic procedures, couples will receive HIV prevention counseling, HIV testing, and the provision of medications that are offered at their clinic (ART and PrEP at clinics that have initiated the intervention; ART at clinics not yet implementing the intervention). Pregnancy and STI testing will be conducted when clinically indicated. In accordance with the Uganda clinical guidelines for PrEP delivery, HIV-negative participants will have rapid HIV tests on a quarterly basis and will be assessed for acute HIV infection prior to PrEP dispensing. PrEP use will be suspended if HIV tests are positive or if acute HIV infection is suspected. In line with Uganda recommendations for public health delivery of PrEP, renal monitoring will occur every 6 months during the first year of PrEP use and annually afterwards, should laboratory diagnostics be available.[33]

At a subset of visits (up to 10%, randomly selected), a plasma sample or dried blood spot will be archived from HIV-1 uninfected partners taking PrEP, for testing including tenofovir levels (as an objective measure of PrEP adherence). Blood samples for tenofovir levels will be shipped to Seattle for testing. This test cannot be conducted in Uganda, because we do not have equipment to perform this specialized testing. We will also extract clinic data from regular CD4 counts and plasma viral loads that are done for HIV-1 infected partners according to local standard of care and support Ugandan clinics to conduct the standard viral load testing 3-6 months after ART initiation.

Procedural tables

HIV-negative partners

	Scr	Enr	M1	M3-M24	Exit
Implementation procedures					
Collect detailed contact/locator information	X			Update annually	
Collect demographic and medical history information	X				
Collect HIV risk information	X		X	X	X
Collect data on fertility desires, contraceptive use, pregnancy		X	X	X	X
Couples-based HIV counseling on HIV prevention, including the strategy of PrEP integrated with ART	X	X	X	X	X
HIV testing and acute HIV screening	X		X	X	X
Condom provision	X		X	X	X
PrEP provision		Aligning with participant preference and HIV risk			
Counseling on PrEP and PrEP adherence		At all visits, aligning with PrEP provision			
Self-report of PrEP adherence			At all visits, aligning with PrEP provision		
Hepatitis B testing	X				
Renal function monitoring	X			6-monthly if using PrEP	
Research procedures					
Informed consent	X	X			
Blood sample collected for archival		X		If part of random selection	X

Behavioral questionnaires:					
Self reported PrEP interruptions				At M3 and then 6-monthly	X
HIV prevention preferences		X		At M3 and then 6-monthly	X
Facilitators and barrier to PrEP use		X		At M3 and then 6-monthly	X
Perception of HIV risk		X		At M3 and then 6-monthly	X
Detailed sexual behavior		X		At M3 and then 6-monthly	X
Internalized stigma		X		At M3 and then 6-monthly	X
Relationship power and intimate partner violence		X		At M3 and then 6-monthly	X
Alcohol use		X		At M3 and then 6-monthly	X
Modeling of PrEP taking behavior		X		At M3 and then 6-monthly	X

HIV-positive partners

	Scr	Enr	M1	M3-M24	Exit
Implementation procedures					
Collect detailed contact/locator information	X			Update annually	
Collect demographic and medical history information	X				
Collect HIV risk information		X			
Collect data on fertility desires, contraceptive use, pregnancy		X	X	X	X
HIV testing	X	X			
Condom provision	X	X	X	X	X
Counseling on ART and ART adherence	X	X	X	X	X
ART provision	X	X	X	X	X
Self-report of ART adherence		X	X	X	X
HIV disease monitoring (Viral load and CD4 count)		X	At M3-6 and thereafter according to natl guidelines		
Couples-based HIV counseling on HIV prevention, including the strategy of PrEP integrated with ART	X	X	X	X	X
Research procedures					
Informed consent	X	X			
Behavioral questionnaires:					
HIV prevention preferences		X		At M3 and then 6-monthly	X
Facilitators and barrier to ART use		X		At M3 and then 6-monthly	X
Self-perceived risk of transmitting HIV		X		At M3 and then 6-monthly	X
Detailed sexual behavior		X		At M3 and then 6-monthly	X
Internalized stigma		X		At M3 and then 6-monthly	X
Relationship power and intimate partner violence		X		At M3 and then 6-monthly	X
Alcohol use		X		At M3 and then 6-monthly	X
Witnessing of PrEP taking behavior		X		At M3 and then 6-monthly	X
Self-efficacy of ART use and influence of PrEP on self-efficacy for ART adherence		X		At M3 and then 6-monthly	X

Retention and exit from research activities

Retention measures will mimic those used with patients enrolled in cliinic ART programs under the standard of care. These include reminder phone calls and text messages, the formation of peer support networks, and visits to people's homes only when they have defaulted from several scheduled visits and if there is an abnormal laboratory result that requires follow up.

Special circumstances

HIV seroconversion

Seroconversion will be determined by local HIV testing guidelines. For initially HIV uninfected participants who seroconvert, a plasma sample or dried blood spot will be collected and archived for tenofovir levels and resistance testing. The index partner will also have a blood sample collected for viral load testing. Couples in which the initially HIV-1 uninfected participant seroconverts will be exited from the study but will continue with their normal HIV clinic care follow up as usual.

Pregnancy

Women who become pregnant while using PrEP will be counseled about the risks and benefits of using PrEP during pregnancy, including the risk of HIV acquisition, according to WHO and U.S. CDC PrEP guidelines.[13, 34] HIV-positive women who become pregnant will be provided with PMTCT services at the clinic.

Relationship dissolution

Couples who break up during the course of follow up will remain in the study but can have separate clinic visits and we will continue to collect data on PrEP, ART use, and HIV risk.

Safety Monitoring

Given the known high safety of antiretroviral medications for both treatment and prevention, and the focus of the project on implementation of PrEP as a bridge to ART rather than the medications themselves, only data on serious adverse events (SAEs) that are unexpected and felt by treating clinicians to be related to the intervention will be collected.

Medications

Medications for PrEP and ART will be dispensed quarterly and will be stored according to the drug manufacturer's recommendations and following standard procedures at the clinics. Adherence counseling is a core part of the PrEP as a bridge to ART intervention and will be done by clinic staff. TDF-containing antiretrovirals are recommended as PrEP by the World Health Organization, including co-formulated FTC/TDF and 3TC/TDF, and the single agent TDF. Any TDF-containing medications that align with WHO and Ugandan national guidelines for PrEP will be used. PrEP will be prescribed for once-daily use. PrEP medication will be provided by Ugandan national stocks of antiretrovirals and/or from donation from Gilead Sciences. PrEP will be dispensed in an amount to last until the next scheduled visit. ART medications will be provided according to Ugandan national ART policies and will come from clinic stocks.

Qualitative interviews and field observations

Providers will be recruited from each clinic for participation in qualitative interviews. At each facility, the local research team will request from the clinic's management, a list of providers with experience providing HIV care, treatment, and/or prevention services. These providers will be approached in person or telephonically by study staff to schedule a time outside of working hours to discuss their study participation and to ascertain their interest and willingness to participate in the study. Interviews with members of HIV serodiscordant couples and providers will be carried out by Ugandan research assistants trained in methods of qualitative interviewing. We anticipate each interview will last 1-1½ hours. Interviews will be conducted in private, neutral locations outside of the clinic where conversations cannot be overheard Interviews may also occur via phone in order to align with social distancing regulations imposed during COVID-19. Interviews will follow pre-piloted topic guides, developed through literature review and our ongoing quantitative research, with flexibility to explore probes and relevant content. Interviews will be recorded, transcribed, and translated into English.

Repeated in depth interviews with HIV serodiscordant couples.

Individual interviews with members of HIV serodiscordant couples will be conducted in the couples' language of preference (English or Luganda). Up to 50 HIV serodiscordant couples will be purposively sampled to ensure that the overall sample includes: a) couples in which the HIV-positive partners have quickly initiated ART and delayed ART initiation, b) couples in which the HIV-negative partners have quickly initiated PrEP and delayed PrEP, and c) gender distribution among HIV-positive and negative partners. Each member of the selected couples will be interviewed separately approximately 3 months after study enrollment. The goal of these interviews will be to elicit information on: 1) facilitators and barriers to ART initiation immediately following an HIV diagnosis, 2) how PrEP use influences ART initiation and 3) perceptions of COVID-19 on PrEP and ART. In conducting interviews, we will attend particularly to exploring the significance of partner gender, relationship dynamics, and intimate partner violence.

Individual follow up interviews with half of the qualitative sample (25 couples) will be conducted to better understand potential barriers to ART initiation and the meaning of PrEP use for a couple. Multiple interviews greatly enhance data quality, as each opportunity for discussion elaborates and sharpens detail, fills gaps in emerging "stories," and allows new topics to be addressed as they come up. The couples sample for follow-up interviews will be selected purposively based on initial interview data, to represent a variety of experiences. A total of up to 150 interviews with members of HIV serodiscordant couples will be carried out for the study (100 early interviews and up to 50 follow up interviews).

In depth interviews with HIV care and treatment providers

Thirty-six (36) HIV care and treatment providers from within the public health clinics participating in the stepped-wedge study will be recruited for in depth interviews that will be conducted in English. The goal of these interviews will be to explore provider opinions about and experiences prescribing PrEP, ART initiation immediately upon HIV diagnosis, perspectives on the integrated PrEP and ART strategy, experiences delivering the intervention and perceptions of COVID-19 on PrEP and ART. Two to three providers from each public health clinic will be recruited. Qualitative interviews will be initiated during step 2 of the stepped-wedge study once multiple clinics have been implementing the intervention. Prior to the initiation of data collection, research assistants will conduct information sessions at the public health clinics describing the study and will invite referral staff to participate.

Field observations

Up to 50 field observations will be conducted at the public health clinics with HIV serodiscordant couples and providers. The purpose of the observations will be to qualitatively describe implementation of the integrated strategy in public health clinics and interactions between members of couples and providers during counseling sessions. Observations will focus on major activities of PrEP and ART delivery: counseling for PrEP and ART initiation and adherence, clinical follow-up appointments; and drug-dispensing at the pharmacy. Each observation session will last two-to-three hours. To minimize bias in collecting observational data, dates of observations will be randomly selected. Observations will be conducted by Ugandan research assistants trained by the qualitative investigators in observational data collection methods. Observations are conducted with verbal consent from the individuals observed and are recorded as field notes. Field notes are narratives that serve as both formal record and basis for analysis of observational data. Field notes will be produced in English by research assistants as Microsoft Word documents.

Microcosting

We will estimate 1) incremental costs (incremental relative to standard practice) for the intervention and 2) treatment costs incurred (and averted) as a result of the intervention. Micro-costing studies at clinics delivering integrated PrEP and ART and the standard of care will be conducted using activity-based approaches for costs incurred (clinic wide trainings, recruitment, service delivery, lab monitoring, PrEP and ART support, and provision of PrEP and ART) and costs averted (health costs saved by averting HIV infections). Cost data will also be collected from the study budget, public health clinic budgets, published government reports, and the health economics literature. Time and motion studies will be conducted by observing visits of HIV serodiscordant couples, and staff time spent on counseling, clinical procedures, and delivering PrEP and/or ART. Adjusting for time spent on research activities (e.g. informed consent, research questionnaires), the total time required for the intervention will be estimated. Through discussions with clinic staff and accounting for time available for the intervention, the time and costs for the delivery of integrated PrEP and ART in public health clinics will be estimated. Importantly, the time spent to deliver services in clinics implementing integrated PrEP and ART will be compared to staff time spent to deliver services in clinics implementing the standard of care. Costs incurred by patients to access care via each of the modalities tested will be assessed through participant interviews. Using estimates of cost from the clinic budget (top-down expenditure analysis) we will account for wastage of other losses.

Intervention fidelity

To monitor intervention fidelity, a member of the training team will make unannounced visits to the clinic to observe counseling sessions. The observer will score each component of the intervention as Not completed/Completed unsatisfactorily/Completed satisfactorily. Additionally, the observer will talk with the couple following the session to get a general sense of whether their counseling experience was similar to previous counseling experiences.

IV. DATA COLLECTION

We will use computer-based or paper-based data collection forms for research variables and link to medical records data captured through standard clinic systems (e.g., routine ART provision, CD4 monitoring). All data will be maintained in a secure location. Internal quality control reports will be run on a monthly basis.

V. DATA ANALYSIS

Analysis of effectiveness of the integrated PrEP and ART intervention

Our primary analyses will use individual-level data from all couples enrolled in the longitudinal cohort. Generalized mixed models with a logistic link will be used to estimate the effect of the intervention on each outcome: a) PrEP initiation, b) PrEP adherence, c) ART initiation, and d) ART adherence. The key predictor in each model is the intervention status of the site at the time the couple was enrolled (delivering standard of care or integrated PrEP and ART), entered as a fixed effect. Models will adjust for "Step" when participant's ART status was assessed as a fixed effect, and will adjust standard errors for correlation of outcomes within clinic by including "clinic" as a random effect.

PrEP as modeled behavior for ART use

We will use descriptive methods to summarize the degree to which PrEP-taking behavior is modeled by HIV negative partners and witnessed by HIV-positive partners. We will use generalized mixed models to determine the degree to which modeled behavior affects the association between the integrated PrEP and ART strategy (randomized exposure) and ART initiation and adherence (as separate outcomes). Models will adjust for the "step" when participant's ART status was assessed as a fixed effect, and will adjust standard errors for correlation of outcomes within clinic by including "clinic" as a random effect. Modeled behavior will be included as a covariate and we will estimate the change in the overall association when the covariate is added.

Analysis of barriers and facilitators of PrEP and ART use

We will use generalized estimating equations to conduct analyses examining factors relating to the use of the integrated PrEP and ART intervention, including fertility desires, estimates of relationship power dynamics, intimate partner violence, alcohol use, gender as key factors. In addition to the key outcomes of PrEP and ART initiation and adherence, we will use a composite outcome of "couple-level HIV prevention" to incorporate the use of PrEP or ART with sufficient adherence to provide HIV protection to a couple. All exposures will be time dependent. Multivariate models will incorporate factors that are associated with couple-level HIV prevention at a p-value <0.10.

Analysis of qualitative interviews and field observations

The goals of the qualitative data analysis are thorough characterization of the meaning of an integrated PrEP and ART strategy for HIV serodiscordant couples and identification and description of implementation challenges encountered by couples and providers. All of the interview transcripts and observation field notes will be included for analysis. Content analysis is our overall analytic approach and we will incorporate four activities: (1) data reduction through coding, (2) category construction, (3) comparison, and (4) interpretation. Our qualitative approach of building in multiple perspectives on topics under investigation creates conditions for triangulation, i.e. validation through cross-validation of emerging results, as an integral part of the analysis.

Estimating cost-effectiveness and budget impact

Using the effectiveness estimated through modeling and the costing from this study, the incremental cost effectiveness ratio (ICER) per incident HIV case and DALY averted will be estimated for the integrated PrEP and ART intervention compared to the standard of care. The primary analysis will be from the programmatic perspective, the purview of decision makers. A secondary analysis will be presented from the societal perspective, using data on economic productivity for HIV disability averted[35] and costs of accessing services. Following WHO guidelines, interventions will be considered cost-effective if the ICER is <3 times local gross domestic product (GDP) and very cost-effective if the ICER is <1 times local GDP per DALY averted.[36] This will facilitate comparison to other strategies and help decision makers

to define priorities and allocate resources.[37] For budget impact analysis, we will consider direct program costs, to ensure that measurements of Ugandan Ministry of Health costs reflect the opportunity cost of the resources used in delivering services. The HIV transmission models, grounded in realistic assumptions of PrEP and ART effectiveness and associated costs, will be a powerful tool for decision makers to estimate the impact of potential prevention interventions. To the extent possible, we will use guidelines to facilitate standardization of cost data collection and reporting and to increase the transparency and generalizability of our results. For all key inputs and outputs, we will follow standard practices,[38] including the guidelines by the Panel of Cost-Effectiveness in Health and Medicine.[39] We will report on all costs using a recommended discount rate of 3% per year, as well as an alternative 5% discount rate and undiscounted inputs

VI. HUMAN SUBJECTS CONSIDERATIONS

The protocol, informed consent forms (for cohort participation and for interviews of providers), and patient education and recruitment materials will be reviewed and approved by the IRBs/ECs responsible for oversight. Subsequent to initial review and approval, the responsible IRBs/ECs will review the study at least annually.

Study oversight

An independent external data monitoring committee will be established to protect patient safety by monitoring study outcomes, implementation, and data quality. The committee will consist of expert clinicians, statisticians, and scientists, including Ugandans, in the field of PrEP and ART delivery in sub-Saharan African settings. Review will be in an unblended fashion, consistent with the open-label, non-randomized nature of the study design. The committee will make recommendations to the study team as part of its six-monthly reviews and reports stemming from these reviews will be provided for submission to overseeing IRBs/ECs.

Informed Consent

We will perform paper-based or electronic-based consenting of participants. After we have ensured that participants have read and understood the consent forms, we will ask them to append their signature on a tablet or on paper. Each participant will receive a participant information sheet that they can use for reference. However, any participant who wants their consent form to take home will have it printed and given to them. Using electronic-based consenting will greatly reduce the burden of storing paper-based consent forms at each of the public health clinics, an activity that would require dedicated safe storage space and staff time for filing. However, if the electronic-based system is not available at a clinic, a paper-based system will be used.

Risks

Partner participants may feel pain or discomfort from phlebotomy if selected for a blood sample archive. Participants may become embarrassed, worried, or anxious when answering behavioral or demographic questions. Although study sites will make every effort to protect participant privacy and confidentiality, it is possible that participants' involvement in the study could become known to others, and that social harms may result (i.e., because participants could become known as participating in a trial involving HIV-1 infected persons). For example, participants could be treated unfairly or discriminated against, or could have problems being accepted by their families and/or communities.

Benefits

Participants accessing care at engaged clinics and part of the cohort will receive individualized and couples HIV risk-reduction counseling and access to PrEP study medication. In addition, participants may benefit in the future from information learned from this study. Providers of HIV care, treatment and prevention who are engaged for in depth interviews may benefit in the future from information learned from this study including novel approaches to provide care for HIV serodiscordant couples. The proposed study will produce information for HIV serodiscordant couples, HIV policy governing bodies, and HIV care, treatment and prevention providers about the implementation of an integrated PrEP and ART strategy in Ugandan public health clinics. This information may have substantial impact on the scale up of PrEP and ART delivery and the global burden of HIV.

Care for persons identified as HIV positive

This study will identify persons who are infected with HIV, either as part of the study screening process or during follow-up of enrolled participants. Study staff will provide participants with their HIV test results in the context of post-test counseling. Persons identified as HIV-infected will be referred for care.

Treatment for injury

Participants will be asked to inform the clinic staff if they feel they have been injured because of taking part in the study. Injuries may also be identified during laboratory testing, medical histories, and physical examinations. Treatment for adverse events related to study participation will be provided by the treatment clinic. If treatment is required that is beyond the capacity of the clinic, the clinic staff will refer the participant to appropriate services or organizations that can provide care for the injury. This study based at IDI will cover the costs of the referral and treatment costs up to the time the participant is stable or the injury is resolved. The participants will be informed of all treatment options available for management of their conditions. The referral process will be documented and will be adhered to as stipulated in national guidelines on referral.

Study records

The Uganda site investigator will maintain, and store in a secure manner, complete, accurate, and current study records throughout the study. Study records include administrative documentation and regulatory documentation as well as documentation related to each participant enrolled in the cohort, including informed consent forms, data forms, notations of all contacts with the participant, and all other source documents.

Confidentiality

Every effort will be made to protect participant privacy and confidentiality to the extent possible. Personal identifying information will be retained at the local study site.

VII. CLINICAL RESEARCH SITE

Site Location

Kampala Site is part of the Infectious Diseases Institute, Makerere University College of Health Sciences. The facility is located next to Kasangati Health Center IV and has adequate space and equipment for ongoing and planned research studies. The Infectious Diseases Institute training team offers specialized courses in HIV management for health providers in Africa. For this study the focus of the training will be combination HIV prevention services including PrEP.

Clinical Facilities

The site has a large training room that can accommodate up to 50 people, 3 clinical rooms, 6 counseling rooms, a pharmacy, a phlebotomy room, a side lab, secure data room with data archive, administrative office space, wireless internet, telephone intercom system, community education offices, and a large waiting area which provide sufficient space for the execution of this study.

Staff

The Principal Investigator is a seasoned HIV/AIDS researcher and trainer, of local and international repute. He will lead the study team to maximize the scientific, ethical integrity of the study and ensure the training provided is of high quality.

The study employs in total 3 doctors, 2 nurse counselors, 1 pharmacy technician, 2 laboratory staff and 2 community educators/counselors, 2 data personnel, 4 qualitative research associates as well as support and administrative staff. The respective staffs are overseen by a site coordinator, training coordinator and administrator. The study site previously implemented the Partners PrEP study and the Partners Demonstration Project. The staff have substantial experience in the use of PrEP and its accompanying supportive services. The staff have been involved in the conduct of protocol specific training, development of IEC materials and have skills in training of adult learners.

Administrative Procedures

All administrative procedures regarding protocol compliance, study coordination, study activation, study monitoring, study records, and use of information and publications will be done according to good clinical practice.

Laboratory Considerations

We will as much as possible use the public health laboratory facilities for routine laboratory monitoring. In situations where this is not possible specimens will be collected and transported to the study collaborating laboratories. All specimen collection, transport, processing, testing, archiving, and results reporting will be conducted in accordance with good clinical and laboratory practice standards. The collaborating laboratories for this study are the MUJHU-IDI Core Laboratory, the Makerere University Walter Reed Laboratory and the Molecular Biology Laboratory in the Department of Medical Microbiology at Makerere College of Health Sciences. These are well-established labs with extensive experience in supporting clinical trials. External Quality Assurance (EQA) procedures will be followed throughout the study, and are overseen by the University of Washington International Clinical Research Center.

VIII. REFERENCES

REFERENCES

[1] UNAIDS. The Gap Report. Geneva, Switzerland2014.
[2] Beyrer C, Birx DL, Bekker LG, et al. The Vancouver Consensus: antiretroviral medicines, medical evidence, and political will. Lancet. 2015;386:505-7.
[3] Dunkle KL, Stephenson R, Karita E, et al. New heterosexually transmitted HIV infections in married or cohabiting couples in urban Zambia and Rwanda: an analysis of survey and clinical data. Lancet. 2008;371:2183-91.
[4] Piot P, Bartos M, Larson H, Zewdie D, Mane P. Coming to terms with complexity: a call to action for HIV prevention. Lancet. 2008;372:845-59.
[5] Ware NC, Wyatt MA, Haberer JE, et al. What's Love Got to Do With It? Explaining Adherence to Oral Antiretroviral Pre-exposure Prophylaxis (PrEP) for HIV Serodiscordant Couples. J Acquir Immune Defic Syndr. 2012.
[6] Ngure K, Mugo N, Celum C, et al. A qualitative study of barriers to consistent condom use among HIV-1 serodiscordant couples in Kenya. AIDS care. 2012;24:509-16.
[7] Ngure K, Shell-Duncan B, Curran K, et al. Following the doctor's advice: Experiences of HIV serodiscordant coulpes enrolled in a PrEP demonstration project in Kenya. Poster 46.06. HIV Research for Prevention. Cape Town2014.
[8] Ware NC, Idoko J, Kaaya S, et al. Explaining adherence success in sub-Saharan Africa: an ethnographic study. PLoS medicine. 2009;6:e11.
[9] Baeten JM, Donnell D, Ndase P, et al. Antiretroviral prophylaxis for HIV prevention in heterosexual men and women. The New England journal of medicine. 2012;367:399-410.
[10] Grant RM, Lama JR, Anderson PL, et al. Preexposure chemoprophylaxis for HIV prevention in men who have sex with men. The New England journal of medicine. 2010;363:2587-99.
[11] Thigpen MC, Kebaabetswe PM, Paxton LA, et al. Antiretroviral preexposure prophylaxis for heterosexual HIV transmission in Botswana. The New England journal of medicine. 2012;367:423-34.
[12] Choopanya K, Martin M, Suntharasamai P, et al. Antiretroviral prophylaxis for HIV infection in injecting drug users in Bangkok, Thailand (the Bangkok Tenofovir Study): a randomised, double-blind, placebo-controlled phase 3 trial. Lancet. 2013;381:2083-90.
[13] World Health Organization. Guideline on When to Start Antiretroviral Therapy and on Pre-Exposure Prophylaxis for HIV. Geneva, Switzerland: World Health Organization; 2015.
[14] South Africa Medicines Control Council. Medicines Control Council approves fixed-dose combination of tenofovir disoproxyl fumarate and emtricitabine for pre-exposure prophylaxis of HIV. 2015.
[15] Cohen MS, Chen YQ, McCauley M, et al. Prevention of HIV-1 infection with early antiretroviral therapy. The New England journal of medicine. 2011;365:493-505.
[16] INSIGHT START Study Group, Lundgren JD, Babiker AG, et al. Initiation of Antiretroviral Therapy in Early Asymptomatic HIV Infection. The New England journal of medicine. 2015;373:795-807.
[17] Cohen M, Chen Y, McCauley M, et al. Antiretroviral Treatment Prevents HIV Transmission: Final Results from the HPTN 052 Randomized Controlled Trial. 8th IAS Conference on HIV Pathogenesis, Treatment and Prevention. Vancouver, Canada2015.
[18] Mujugira A, Celum C, Thomas KK, et al. Delay of antiretroviral therapy initiation is common in East African HIV-infected individuals in serodiscordant partnerships. Journal of acquired immune deficiency syndromes (1999). 2014;66:436-42.

[19] Siedner MJ, Santorino D, Lankowski AJ, et al. A combination SMS and transportation reimbursement intervention to improve HIV care following abnormal CD4 test results in rural Uganda: a prospective observational cohort study. BMC Med. 2015;13:160.
[20] Rosen S, Fox MP. Retention in HIV care between testing and treatment in sub-Saharan Africa: a systematic review. PLoS medicine. 2011;8:e1001056.
[21] Katz IT, Essien T, Marinda ET, et al. Antiretroviral therapy refusal among newly diagnosed HIV-infected adults. AIDS (London, England). 2011;25:2177-81.
[22] Curran K, Ngure K, Shell-Duncan B, et al. 'If I am given antiretrovirals I will think I am nearing the grave': Kenyan HIV serodiscordant couples' attitudes regarding early initiation of antiretroviral therapy. AIDS (London, England). 2014;28:227-33.
[23] Govindasamy D, Ford N, Kranzer K. Risk factors, barriers and facilitators for linkage to antiretroviral therapy care: a systematic review. AIDS (London, England). 2012;26:2059-67.
[24] Remien RH, Bauman LJ, Mantell JE, et al. Barriers and facilitators to engagement of vulnerable populations in HIV primary care in New York City. Journal of acquired immune deficiency syndromes (1999). 2015;69 Suppl 1:S16-24.
[25] Baeten JM, Heffron R, Kidoguchi L, et al. Integrated delivery of antiretroviral treatment and pre-exposure prophylaxis to HIV-1-serodiscordant couples: A prospective implementation study in Kenya and Uganda. PLoS medicine. 2016;13:e1002099.
[26] Bandura A. Social Learning Theory Prentice Hall; 1976.
[27] Bandura A. Self-efficacy: toward a unifying theory of behavioral change. Psychol Rev. 1977;84:191-215.
[28] Simoni JM, Nelson KM, Franks JC, Yard SS, Lehavot K. Are peer interventions for HIV efficacious? A systematic review. AIDS and behavior. 2011;15:1589-95.
[29] Jones D, Cook R, Spence A, Weiss SM, Chitalu N. Antiretroviral therapy in Zambia: do partners on ART enhance adherence? J Int Assoc Provid AIDS Care. 2014;13:497-500.
[30] Remien RH, Stirratt MJ, Dolezal C, et al. Couple-focused support to improve HIV medication adherence: a randomized controlled trial. AIDS (London, England). 2005;19:807-14.
[31] Baeten JM, Donnell D, Mugo NR, et al. Single-agent tenofovir versus combination emtricitabine plus tenofovir for pre-exposure prophylaxis for HIV-1 acquisition: an update of data from a randomised, double-blind, phase 3 trial. The Lancet infectious diseases. 2014;14:1055-64.
[32] Curran K, Baeten JM, Coates TJ, Kurth A, Mugo NR, Celum C. HIV-1 prevention for HIV-1 serodiscordant couples. Current HIV/AIDS reports. 2012;9:160-70.
[33] Ministry of Health RoU, ,. Technical Guidance on Pre-Exposure Prophylaxis (PrEP) for Persons at High Risk of HIV in Uganda. Kampala, Uganda: Ministry of Health; 2016.
[34] U. S. Public Health Service. Pre-exposure prophylaxis for the prevention of HIV infection in the United States - 2014. 2014 Clinical Practice Guideline. 2014.
[35] Murray CJL, Lopez AD, Harvard School of Public Health., World Health Organization., World Bank. The global burden of disease : a comprehensive assessment of mortality and disability from diseases, injuries, and risk factors in 1990 and projected to 2020. Cambridge, MA: Published by the Harvard School of Public Health on behalf of the World Health Organization and the World Bank ; Distributed by Harvard University Press; 1996.
[36] UNAIDS. Costing Guidelines for HIV Prevention Strategies. Geneva: UNAIDS; 2000.
[37] Galarraga O, Colchero MA, Wamai RG, Bertozzi SM. HIV prevention cost-effectiveness: a systematic review. BMC public health. 2009;9 Suppl 1:S5.

[38] Drummond M, Drummond M. Methods for the economic evaluation of health care programmes. 3rd ed. Oxford ; New York: Oxford University Press; 2005.
[39] Gold MR. Cost-effectiveness in health and medicine. New York: Oxford University Press; 1996.
