# Supplementary material for: How PrEP delivery was integrated into public ART clinics in central Uganda: A qualitative analysis of implementation processes
Source: PLOS Glob Public Health. 2024 Mar 7;4(3):e0002916. doi: 10.1371/journal.pgph.0002916 (PMC10919847; doi:10.1371/journal.pgph.0002916)
Supplement: S2 File — This qualitative interview guide was used for initial interviews with partners living with HIV who were taking ART. (PDF) [file pgph.0002916.s003.pdf]

Partners PrEP Program Qualitative Interview Guide
Index Participants (Partners Living with HIV)
Version 2.0 – July 25, 2019

Introduction
I am talking to you because you are a research participant in the Partners PrEP Program. You and your partner/ spouse are in a serodiscordant relationship, in which one of you is HIV-positive and the other is HIV-negative. During our conversation today, we will talk about your relationship with your partner. We will also discuss you and your partner's experiences receiving medications for HIV treatment and prevention. What you tell me is confidential and will not be shared with anyone you know. Do you have any questions before we begin?

Background
Let's get started by you telling me a little about yourself.

In which community do you live?
Who do you live with?
Tell me about your family (children, spouse, girlfriend/ boyfriend).

How do you feel about your home/ living situation?

What activities do you do to generate income? Tell me a little about these activities.

What kind of financial support do you give to/ get from your primary partner?

Relationship
I would first like to talk to you about your relationship with the partner with whom you joined PPP. Tell me about your relationship.

Are you still together as a couple?

<u>Note</u>*: If the couple has separated, you must adapt the questions in the following section to learn about the relationship when they were together. (Questions should be phrased in the past tense.)*

What happened to cause you to separate? Tell me about this.

Probe in detail.

How long have you been (were you) together?

Do you stay together – i.e., live and sleep in the same household?
Do you have children together?
Do you plan to have more children? Tell me about this.

How would you describe your relationship currently?

What is your life like together on a day-to-day basis (if applicable)?

In what ways do you admire/ respect your partner?

Probe in depth.

Do you have any concerns about your relationship? What are they?

Are you ever worried that your partner may mistreat you? Tell me about that.

Discovery of HIV Status

Now I would like for you to tell me the story of how you first learned you were HIV-positive.

Where did you test? When did this happen?

What happened after testing?

How did you feel about your results?

What happened when you learned you and the partner you joined PPP with were serodiscordant?

Probe in detail.

In what ways did serodiscordance affect/ change your relationship?

ART Initiation

Tell me the story of what happened when you were first offered ART.

When and where did you initiate ART? *Probe in detail.*

What were you reason(s) for starting ART?

In your opinion, what was most important in encouraging you to start ART?

What were your concerns about starting ART?

Probe to understand any reluctance, delays and/or refusal to initiate ART.

Clinic Experiences

Where do you go for your health care currently?

How did you end up going to _____________ *(name of clinic)*?

Probe in depth for the story. If this clinic is different than the PPP clinic, probe to find out why.

What were the reasons you chose to go to _____________?

As part of the PPP, serodiscordant couples are offered pre-exposure prophylaxis (PrEP) for the negative partner and ART for the positive partner. How did you learn that PrEP and ART were available at the clinic?

Probe in depth for the story.

Was the availability of ART and PrEP a factor in choosing _____ Health Centre? Why?

What had you heard about PrEP before going to the clinic, if anything?

What were you counseled at the clinic about taking PrEP and ART together as a couple?

What did you understand to be the purpose of taking these medications?

PrEP Initiation

We are now going to talk about your (former) partner. Does your (former) partner go to the same clinic as you? What services does s/he receive there?

What do you know about what happened when s/he was offered PrEP at the clinic?
Probe in depth for the story.

Did s/he end up starting PrEP? Tell me the story of what happened.
Probe in depth.

How did you feel about him/her starting/ not starting PrEP?

What did you understand were the reasons your partner wanted/did not want to take PrEP?
Probe in depth.

What concerns did your partner express about taking PrEP?
Explore any reluctance and/or delays in starting PrEP.

Is your (former) partner still taking PrEP?
If yes: How has that been for him/her?
If no: Tell me about him/ her discontinuing PrEP. What happened?
What were his/her reasons for stopping?
Probe in depth.

ART and PrEP Use/ Adherence

You said you initiated ARVs in _________. How long have you been taking ARVs? How has it been for you, generally?

How do you usually take your antiretrovirals?
Probe for a "typical" story about how pills are taken – i.e., when, where, in the presence of whom, etc.

What happens when you go to the clinic to collect your ART refills?

Does your partner go to the clinic together with you?
Tell me a story about how that works.

What does your partner do to support you in taking medication?
Give me an example of what you mean by _____________.

Have you and your partner ever taken your doses together?
If yes: Tell me about the last time you took ART at the same time your partner took PrEP. What happened?

How does it make you feel when you take your doses together?

If not: Would you like to take your doses together? Tell me about this.

What does taking ART and PrEP do for your relationship with your partner?
How has taking medication been good for your relationship?
Are there ways it has been bad? What are they?

Some people find it difficult to swallow their pills all the time. Tell me about a recent time when you did not take your ARVs.
Possible probes:
What happens with your pills when…
…other people are around you when it's time to take your pills?
… you are not home at the dosing time?
… you take alcohol?

What does your partner do when you miss taking your pills?
Probe in depth.

How do you feel about having to take ARVs every day for the rest of your life?
Probe the response.

What else should I know about your experiences taking ARVs for HIV?

Closing

As a participant in the PPP research study, you also take part in separate study visits. Tell me about these visits.

What were you reasons for deciding to join the PPP research study?

How has it been for you to participate in the PPP?

The PPP is investigating couples' experiences taking ARVs for treatment and for prevention at the same time? What has this meant for you personally?
Probe in depth.

Is there anything else I should know in order to understand you and your partner's experiences with PrEP and ART?
