# Supplementary material for: How PrEP delivery was integrated into public ART clinics in central Uganda: A qualitative analysis of implementation processes
Source: PLOS Glob Public Health. 2024 Mar 7;4(3):e0002916. doi: 10.1371/journal.pgph.0002916 (PMC10919847; doi:10.1371/journal.pgph.0002916)
Supplement: S3 File — This interview guide was used for initial interviews with HIV-negative partners who initiated PrEP. (PDF) [file pgph.0002916.s004.pdf]

Partners PrEP Program Qualitative Interview Guide
Partner Participants
Version 2.0 – July 25, 2019

Introduction
I am talking to you because you are a research participant in the Partners PrEP Program. You and your partner/ spouse are in a serodiscordant relationship, in which one of you is HIV-positive and the other is HIV-negative. During our conversation today, we will talk about your relationship with your partner. We will also discuss you and your partner's experiences receiving medications for HIV treatment and prevention. What you tell me is confidential and will not be shared with anyone you know. Do you have any questions before we begin?

Background
Let's get started by you telling me a little about yourself.

In which community do you live?
Who do you live with?
Tell me about your family (children, spouse, girlfriend/ boyfriend).

How do you feel about your home/ living situation?

What activities do you do to generate income? Tell me a little about these activities.

What kind of financial support do you give to/ get from your primary partner?

Relationship
I would first like to talk to you about your relationship with the partner with whom you joined PPP. Tell me about your relationship.

Are you still together as a couple?

<u>Note</u>*: If the couple has separated, you must adapt the questions in the following section to learn about the relationship when they were together. (Questions should be phrased in the past tense.)*

What happened to cause you to separate? Tell me about this.

Probe in detail.

How long have you been (were you) together?

Do you stay together – i.e., live and sleep in the same household?
Do you have children together?
Do you plan to have more children? Tell me about this.

Tell me the story of how you learned about you and your partner were serodiscordant.

Probe in detail about what happened.

What happened in your relationship after you discovered you and your partner are serodiscordant?

How would you describe your relationship currently?

What is your life like together on a day-to-day basis?

In what ways do you admire/ respect your partner?
Probe in depth.

Do you have any concerns about your relationship? What are they?
Are you ever worried that your partner may mistreat you? Tell me about that.

Clinic Experiences

Where do you go for your health care? Does your (former) partner go to the same clinic?
How did you end up going to _____________ *(name of clinic)*?
Probe in depth for the story of how the couple ended up at the clinic. If the partners go to different clinics now, probe.

What were the reasons you chose to go to _____________?

How did you learn that pre-exposure prophylaxis (PrEP) and antiretroviral treatment (ART) was available at the clinic?
Probe in depth for the story.

Was the availability of ART and PrEP a factor in choosing _____ Health Centre? Why?

What had you heard about PrEP before going to the clinic, if anything?

What were you counseled at the clinic about taking PrEP and ART together as a couple?
What did you understand to be the purpose of taking these medications?

PrEPInitiation

What happened when you were offered PrEP at the clinic?
Probe in depth for the story.

How did you feel about being offered the opportunity to take PrEP?
Probe.

What were you counseled about PrEP?

Did you end up starting PrEP? Tell me the story of what happened.
Note: Some participants have declined PrEP. Probe to understand their reasons for declining. Others may have accepted the pills but do not take them.

What were your reasons for (not) wanting to take PrEP?
Probe in depth. Possible probes: feeling at risk for HIV, hope for staying together, support one's partner, safer conception, inability to negotiate condom use, extra protection, not feeling at risk, fears about PrEP.

Is there anything else that made you (not) interested in taking PrEP?
Tell me about __________.

What fears or concerns did you have about taking PrEP?

Explore any reluctance and/or delays in starting PrEP.

*If s/he did not initiate PrEP:*What would make you interested in taking PrEP in the future? Why?

ART Initiation

We are now going to talk about your partner (the person you joined PPP with). Tell me what you know about what happened when your partner was offered ART.

Did s/he initiate ART? Where and when was this? *Probe in depth.*

Did your partner have any concerns about starting ART? What were they?

Probe to understand any reluctance, delays and/or refusal to initiate ART.

In your opinion, what encouraged your partner to start ART?

How do you think your decision to(not) takePrEP influenced your partner's decision to (not) initiate ART?

Probe to understand the relationship between the PrEP initiation and ART use.

Possible probes: hope for staying together, desire to keep each other healthy, wish to support one's partner

PrEP Use/ Adherence

Note: If participant never initiated PrEP, skip to Closing section. If you learn the individual has stopped PrEP, ask the questions in this section and then move to the next section on PrEP discontinuation.

When did you first start PrEP? Are you still taking PrEP?

Participants who are still taking PrEP only:

How often do you go to the clinic to collect PrEP? What happened at your most recent visit when you collected refills?

Do you and your partner go to the clinic together or separately?

Tell me more about how that works.

How do/didyou usually take your PrEP pills (when you were taking them)?

Probe for a "typical" story about how pills are taken – i.e., when, where, in the presence of whom, etc.

What do/did you and your partner do to support each other in taking your medication?

Give me an example of what you mean by _____________.

Do/did you and your partnerever take your doses together?

Tell me about the last time you took PrEP at the same time your partner took ART. What happened?

How does/did it make you feel when you take/took your doses together?

If not: Would you like to take your doses together? Tell me about this.

What does/did taking PrEP do for your relationship with your partner?
How has taking medication been good for your relationship?
Are there ways it has been bad? What are they?

Some people find it difficult to swallow their pills. Tell me about a (recent time) when you did not take your PrEP.
What happened?

For participants who are still taking PrEP only:
How long do you plan to take PrEP? Why do you say this?

How do you feel about the idea of stopping PrEP eventually?

What will happen when you stop?
In what ways will discontinuing PrEP affect you and your partner?

What else should I know about your experiences taking PrEP for prevention?

PrEP Discontinuation

After you were initiated on PrEP, did you ever go back to the clinic to collect PrEP refills? What happened when you went to the clinic for the refill?

If not:What were your reasons for not going back for refills?

I'd like to ask you some questions about stopping PrEP. How long did you take PrEP before you stopped?

Why did you decide to discontinue taking PrEP?

Was taking PrEP what you expected it to be? What would you have liked to be different?

Can you imagine a time in thefuture when you would take PrEP again? What would make this possible?

Are you still going to the clinic? What happened at your most recent visit?

Do you and your partner go to the clinic together or separately?
Tell me more about how that works.

Closing

As a participant in the PPP research study, you also have separate study visits. Tell me about these visits.

What were you reasons for deciding to join the PPP research study?

How has it been for you to participate in the PPP?

The PPP is investigating couples' experiencestaking antiretrovirals for treatment and for prevention at the same time. What has this meant for you, personally?

Probe in depth.

Is there anything else I should know in order to understand you and your partner's experiences with PrEP and ART?
