# Supplementary material for: How PrEP delivery was integrated into public ART clinics in central Uganda: A qualitative analysis of implementation processes
Source: PLOS Glob Public Health. 2024 Mar 7;4(3):e0002916. doi: 10.1371/journal.pgph.0002916 (PMC10919847; doi:10.1371/journal.pgph.0002916)
Supplement: S4 File — This interview guide was used for follow-up interviews with a subsample of participants living with HIV and their HIV-negative partners. (PDF) [file pgph.0002916.s005.pdf]

Partners PrEP Program Qualitative Interview Guide
Index and Partner Participants (Follow Up Interviews)

Introduction

Thank you for being available to speak with me today. As we discussed when we scheduled this call, we are conducting interviews over the phone instead of in-person. Our last interview was in __________ [month/ year], when we talked about your experiences initiating and taking [ART/ PrEP] as well as your relationship with your partner. The purpose of today's conversation is to get an update on your HIV treatment/ prevention experiences, and your participation in PPP. I will also ask you some questions about your health care experiences in light of the current coronavirus situation in Uganda. Do you have questions?

Partners PrEP Program/ Health Care Experiences

I am going to start by asking you some general questions about your participation in the Partners PrEP Program (PPP) research study at _______ Health Centre/ Hospital. Are you still attending __________ (facility) for your HIV prevention/ treatment services?

> *If not:* Where are you going now? Why did you change facilities (or stop going)?

What can you tell me about PPP? What is the purpose of the program?

PPP uses a strategy of offering antiretrovirals to couples for both HIV treatment and HIV prevention. How well do you think this strategy has been implemented at _________ (facility)? Why?

> What, in your opinion, could be improved about the program?
>
> > *Probe in depth.*

What were you reasons for deciding to join PPP?

Overall, what have you liked personally about PPP?

> What have you found challenging about participating in PPP?

In your opinion, what can you say about how couples-based HIV prevention and treatment services are organized at ___________ (facility)?

> Do you feel these services meet your and your partner's needs? How so?

As part of PPP, you also see health workers from IDI Kasangati either at your facility or at Kasangati site. Are you still seeing these health workers?

> If yes, have you done any "virtual" or phone-based meetings? How did they go?

ART and PrEP Use Experiences

When we first met in [_____ month/ year], you and your partner had recently started taking ART/ PrEP from ____________ (facility). Thinking back to that time, what do you remember about your reasons for initiating ART/ PrEP?

> In our last conversation, you said ___________. Can you explain your thoughts about this now? In what ways have your reasons for taking ART/ PrEP changed over time?

Reflect on what was discussed in first interview, and probe for changes/ inconsistencies.

Are you still taking ART/ PrEP now? How is that going for you, generally?

How would you explain your reasons for taking ART/ PrEP now?

If discontinued: What were the reasons you stopped taking ART/ PrEP? Please tell me the story of discontinuing ART/ PrEP in detail.

Probe about PrEP stop.

If no longer taking medicine: What do you do if you have a health problem nowadays? Have you had to go to a health center in the past few weeks? What happened?

What happened the last time you collected your medicines?

How did you get to the clinic? Is this the same or different as before? How so? What happened when you got there?

Probe in depth about process of collecting your refills since the coronavirus/ national shutdown.

What are your concerns about obtaining refills of your medicines these days (if applicable)?

Probe about worries about resupply, drug shortages and stockouts during the nationwide shutdown.

Since our last interview, has anything changed about the way you take your medicine?

In what ways has it changed?

Probe about new adherence challenges, especially since coronavirus.

Overall, what have been/were some of the major challenges you face(d) in taking ART/ PrEP? Tell me about a specific time when you faced _________ (this challenge).

In the last interview, you said __________________. How has this changed?

What are your plans for taking ART/ PrEP in the future?

If taking PrEP: how will you feel about stopping PrEP? Why?

What does taking ART/ PrEP mean for your HIV prevention?

Aside from ART/ PrEP, what are you doing to protect your partner/ yourself from acquiring HIV?

How do you feel about your/ your partner’s risk for acquiring HIV? *Probe.*

What else should I know about your experiences taking ARVs/ PrEP for HIV treatment/ prevention?

Perspectives on Coronavirus/ COVID-19

We have been hearing a lot about the coronavirus lately, so I would like to get your perspective on the current situation. Would that be ok with you?

What is the coronavirus/ COVID-19?

What do you think when you hear the term “coronavirus” or “COVID-19”?

What are people in your community saying about it?

Tell me about any stigma associated with the virus.

What are people doing when they are experiencing symptoms of COVID-19, or they think they may have been exposed to the virus?

Probe about: access to testing, fears of going to health facilities, concerns about testing process

What guidance has the government given to the public about the coronavirus?

How do you feel about the measures the government has taken to prevent the spread of COVID-19?

Probe about: movement restrictions, social distancing measures, nighttime curfews, closing of businesses

In general, how has the [situation with] coronavirus affected your daily life?

What is it like for you to move around (transportation)?
How has it impacted your ability to go to work or earn income?
In what ways has it affected your ability to access food and other daily necessities?
What is life like at home?

Probe and ask for examples/ stories.

What are your biggest concerns right now?

Probe about financial problems, food insecurity, relationship issues, gender-based violence, lack of privacy at home, cramped living quarters, etc.

How do you feel about this situation, in general?

Probe about the impact on mental health and wellbeing.

What steps are you taking to keep yourself and your family safe?

How helpful are these measures, in your view?

Has COVID-19 illness affected you or your family personally? How?

What has that been like?

What have you heard about treatments for coronavirus COVID-19, if anything?

What is the ____________ (PPP facility) where you get your HIV treatment/ prevention services doing about the coronavirus, as far as you can tell?

Have the services being offered changed or been modified at the clinic since COVID-19? How so?

How did you feel about being at the clinic with other clients during this time of social distancing (if applicable)?

Relationship

I would now like you to think about your relationship with the partner with whom you joined PPP. Are you still together as a couple?

In our last conversation, you told me that you [do/ do not] stay together (live and sleep in the same household). Is this still the case?

How has this been impacted by the transportation and movement restrictions?

How has your relationship changed since we last spoke?

Probe in detail about any changes, including separation or reconciliation. Pay attention to references to intimate partner violence (IPV), and probe, if appropriate.

What were the circumstances around your separation/ reconciliation/ __________? What happened?

If PPP couple has separated, ask about new relationships

How has the coronavirus situation (i.e., staying at home) affected your relationship(s)?

What challenges are you facing? What are you doing to cope with these challenges?

Probe about additional stressors on the relationship.

Do you have any concerns about your relationship? What are they?

During our last conversation, you told me __________________. Is this still the case?

What are your thoughts these days about serodiscordance?

When we spoke last time, you mentioned _____________. Can you say something about how this has changed, if at all?

In our last conversation, you told me you [decided to take a break from sex with your partner/ were continuing sex with your partner]. What are you doing now?

What did the health workers counsel you about sex with your partner?

What are your thoughts about having sex while taking ART/ PrEP? Why do you say this?

Last time, you told me you [were/ were not using] condoms with your partner while [you/ they] are taking PrEP. What are your thoughts about this now?

If applicable: When we spoke last time, you/ your wife was pregnant (or had recently delivered). How are you receiving care for your baby at this time?

Has this changed during the coronavirus situation? In what ways?

ART/ PrEP in the Relationship

You mentioned in the first interview that your (former) partner initiated ART/ PrEP.
Is s/he still taking ART/ PrEP, to your knowledge?

> *If no:* Tell me about him/ her discontinuing ART/ PrEP. What happened?
>
> What is your perspective on how well s/he takes ART/ PrEP?

How do/did you feel about your partner taking ART/ PrEP? Why?

Have you/ did you and your partner ever taken your ART and PrEP together?

> *If yes:* Tell me about a time when you both took your took medicines for HIV prevention and treatment together. What happened?
>
> How does it make you feel when you take your doses together?
>
> *If not:* Would you like to take your doses together? Tell me about this.
>
> > *Probe about changes from first interview.*

What does taking ART and PrEP do for your relationship with your partner?

> How has taking medication been good for your relationship?
> Are there ways it has been bad? What are they?
>
> *If applicable:* How has your relationship changed since your partner stopped taking ART/ PrEP? In what ways?
>
> > *Probe in depth.*

Closing

I just now have a few more questions. Thinking back over our conversation today, would you say the coronavirus/ COVID-19 is a bigger or a smaller concern for you than HIV treatment and prevention? How so?

How would you describe what COVID-19 has meant for your life and your family, overall?

Usually, we do interviews in-person. How did you feel about having this conversation on the phone?

> What were your concerns about us having this conversation by phone?

As we discussed earlier, PPP is investigating couples' experiences taking ARVs for both treatment and for prevention. What has this meant for you, personally?

> *Probe in depth.*

Is there anything else I should know in order to understand you and your partner's experiences with HIV treatment and prevention during this time of COVID-19?

Thank you for sharing your experiences and time with me.
