# Supplementary material for: How PrEP delivery was integrated into public ART clinics in central Uganda: A qualitative analysis of implementation processes
Source: PLOS Glob Public Health. 2024 Mar 7;4(3):e0002916. doi: 10.1371/journal.pgph.0002916 (PMC10919847; doi:10.1371/journal.pgph.0002916)
Supplement: S5 File — This interview guide was used for interviews with health care providers from public health facilities implementing integrated ART and PrEP delivery. (PDF) [file pgph.0002916.s006.pdf]

Partners PrEP Program Qualitative Interview Guide
Provider Participants

Introduction
Thank you for being available to speak with me today. As we discussed when we scheduled this call, we are conducting interviews over the phone instead of in-person. The purpose of this conversation is to discuss your experiences and perspectives providing ART and pre-exposure prophylaxis (PrEP) to serodiscordant couples. I will also ask you some questions about your experiences delivering health care, especially in the context of the current coronavirus situation in Uganda. Whatever we discuss is confidential and will not be shared with your employer or anyone else you know. Do you have any questions before we begin?

Partners PrEP Program at the Facility
Let's get started by telling me a little about yourself. What is your role at ______ health center/ hospital? [Specify PPP facility]

What do you do in a typical work day at your facility?

I understand you are involved in providing PrEP at your site. How are you involved with PrEP, exactly?

When did your facility start providing PrEP?

From which organization did the facility receive PrEP initially? (Partners PrEP Program or PEPFAR?)

When did PPP begin providing PrEP? When did PEPFAR start providing PrEP?

What is your understanding about the Partners PrEP Program?

Why is PPP being implemented at your facility?

What does it do?

What was it like for the facility to incorporate PrEP into existing HIV services?

How difficult was it?

Probe for examples.

How open, or receptive, was your facility to including PrEP as part of your package of HIV prevention services?

Probe in depth.

How does provision of PrEP to HIV-negative persons compare to other priorities for services at your facility?

What can you say about how well PPP "fits" into your HIV program?

What was it like for you and your colleagues (other health care workers) to adapt to providing PrEP?

What "extra" work do you do as part of PPP? How do you feel about this?

How well do you think PPP meets the needs of your clients at ____________?

In what ways does it (not) meet their needs?

How does PPP compare to PEPFAR at your facility?

What are the advantages of PPP?

What are the disadvantages?

Probe about: participant recruitment, time/ space for offering PrEP services, staff capacity to support PrEP service provision, delays in receiving laboratory results

Generally, how has it been managing two different programs providing PrEP?

What are some of the challenges?

What is working well?

Training and Support

Tell me about the training you received to provide PrEP at your facility.

Probe to understand what was provided as part of PPP vs. PEPFAR.

How do you feel about the training you received?

Did the training prepare you to offer PrEP? How so?

What suggestions do you have to improve the training?

What was the most important thing you learned about PrEP?

In addition to training, there is a technical advisory team that is part of PPP. What do the technical advisors (TAs) do?

What feedback do you get from the TAs?

Do they share the TA Reports with you? Tell me about this.

What support do the TAs give you in providing PrEP?

Probe about support, beyond financial incentives, including TA reports, What's App group, etc.

What support does your facility, or your supervisors, give to enable you to provide PrEP?

What would improve the support you get to deliver PrEP at your facility? How so?

How confident do you feel about your ability to provide PrEP?

Probe in depth.

Experiences with PrEP and ART Delivery

I would now like for us to talk about your experiences delivering PrEP. What is the process for clients to receive PrEP at your facility?

How does the clinic determine which clients are (eligible to be) offered PrEP?

Aside from serodiscordant couples, which other people are offered PrEP at your facility?

What do you think of PrEP being made available to these groups?

What is different between serodiscordant couples and other groups who receive PrEP at your facility?

Have you ever had any serodiscordant couples refuse PrEP or ART? What happened?

What do you counsel couples about taking PrEP and ART?

What do you tell them about using condoms while taking PrEP?
Why do you tell them ___________?
How do couples respond to what you tell them?

We've heard from some couples that they are told to take a break from having sex when they are starting PrEP. What do you counsel couples about PrEP and sex?

What do you tell a pregnant woman about PrEP?

What counseling do you give about stopping or discontinuing PrEP?

What do you hear from serodiscordant couples about challenges in taking ART and PrEP for prevention, generally?

In general, what challenges do you face in delivering PrEP and ART to serodiscordant couples?

Perspectives on the Integrated Strategy of PrEP and ART

What is your opinion about the strategy of providing ART to HIV-positive individuals and PrEP to their HIV-negative partners for at least six months after ART has been initiated?

What do you understand about U=U (undetectable = untransmissible)?

How do you think couples understand the concept that ART taken by an HIV-positive person can prevent HIV acquisition in an HIV-negative partner (U=U)?
What would make it easier for them to understand this idea?
Probe in depth.

In what ways does offering PrEP to HIV-negative members of serodiscordant couples help the HIV-positive partner take ART? Do you have any specific examples to demonstrate this?
Probe in depth.

Perspectives on Coronavirus/ COVID-19

We have been hearing a lot about the coronavirus lately, so I would like to get your perspective on the current situation. Would that be ok with you?

What is your understanding of the coronavirus/ COVID-19?
What do you think when you hear the term “coronavirus” or “COVID-19”?

What are people in the community saying about the coronavirus?
Tell me about the stigma associated with the virus.

In what ways has your job function/ work changed since the coronavirus has come to Uganda and there has been a national shutdown?

  What new challenges do you face?

What concerns do you have about being at work right now?

  How do you manage/ cope with these concerns?

What steps has your facility taken to handle the coronavirus situation?

  How do you feel about what the facility has done?

  What fears do you have about being exposed to coronavirus at work?

What is it like for you to get to the health facility, with the transportation restrictions in place?

  Tell me what you do to get to work. In what ways has it changed?

How have the way HIV services are delivered at your facility changed since coronavirus?

  Probe about community-based ART pick up points, multiple-month appointments, etc.

What are some of the challenges your clients are facing with HIV prevention and treatment these days?

  What has happened with access to PrEP and ART during the coronavirus?

  Have you noticed any changes? What are they?

    Probe about concerns about resupply, drug shortages and stockouts during the nationwide shutdown.

In your opinion, how important is HIV prevention to your clients/ patients as compared to concerns about the coronavirus? Why do you say this?

What are your clients saying about their biggest worries or concerns in their lives right now?

  Probe about financial problems, food insecurity, relationship issues, gender-based violence, lack of privacy at home, cramped living quarters, etc.

What worries do your clients have about the coronavirus itself? How do you respond?

Are your clients talking to you about treatment for coronavirus? What do you say?

  What have you heard about treatments for coronavirus/ COVID-19, if anything?

What are people doing when they are experiencing symptoms of COVID-19, or they think they may have been exposed to the virus?

  What do people do if they want to be tested for the coronavirus?

  Probe about: access to testing, fears of going to health facilities, concerns about testing process

What guidance has the government given to the public about the coronavirus?

How do you feel about the measures the government has taken to prevent the spread of COVID-19?

Probe about: movement restrictions, social distancing measures, nighttime curfews, closing of businesses

Has COVID-19 illness affected you or your family personally? How?

What has that been like?

Closing

I just now have a few more questions. How would you describe what COVID-19 has meant for your life, overall?

What are your biggest concerns right now?

How do you feel about this situation, in general?

Probe about the impact on mental health and wellbeing.

Thinking back over our conversation today, how is the coronavirus/ COVID-19 affecting HIV treatment and prevention services in Uganda?

Do you have any final thoughts about delivering ART and PrEP to serodiscordant couples at your facility?

Thank you for your time and participation.
