# Supplementary material for: How PrEP delivery was integrated into public ART clinics in central Uganda: A qualitative analysis of implementation processes
Source: PLOS Glob Public Health. 2024 Mar 7;4(3):e0002916. doi: 10.1371/journal.pgph.0002916 (PMC10919847; doi:10.1371/journal.pgph.0002916)
Supplement: S6 File — This codebook was used to guide the coding process. Codes were derived inductively from interview data. (PDF) [file pgph.0002916.s007.pdf]

PPP Codebook – Version 2.0
Revised 4/7/21

Health Care Experiences and PPP Codes

Counseling – ART Initiation
Accounts of information or instructions shared by health care workers about initiating ART, including explanations about the health benefits of ART and the importance of initiating ART as soon as possible.

Counseling – Adherence
Examples of health care workers providing information, instructions and/or encouragement to support appropriate and effective pill-taking behavior among couples. This may include recommendations that partners monitor ART or PrEP use.

Counseling – PrEP Initiation
Accounts of information or instructions shared by health care workers about initiating PrEP, including explanations about the protective benefits of PrEP.

Counseling – Relationship
Examples of health care workers encouraging couples to remain together and support each other in spite of serodiscordance or other conflicts in the relationship.

Counseling – Risk
Accounts of health care worker messages about risk or risk reduction, especially condom use, including the period in which condoms must be used following PrEP initiation (i.e., typically seven days).

Joining PPP
Couples' reasons or motivations for joining the PPP study. Reasons for joining the study include the opportunity to take PrEP, the perception they will receive better health services through PPP as compared to other facilities, and the hope that the study will benefit them or other serodiscordant couples in the future.

Joint visits
Examples of partners receiving care together and/or descriptions of the ways in which they value couples-focused services. This includes explanations about the importance of partners accompanying each other to clinic visits and how this helps to support their own care.

Missed visits
Descriptions of the reasons for and consequences of missed clinic appointments (to collect medication refills) or PPP study visits.

PPP Clinic Complaints
Negative experiences receiving HIV prevention and treatment at the PPP site and/or dissatisfaction with services. Examples include: inconvenience (single month dispensing of drugs), medication or HIV test stockouts, slow service, long lines, inadequate counseling, incomplete viral load results, the need to go to multiple clinics for care, etc. This includes partners' experiences as well.

PPP Purpose

General thoughts about or perspectives on the purpose of PPP and/or the integrated strategy of ART and PrEP delivery- i.e., explanations of how the PPP study was instrumental in keeping relationships together following serodiscordance. This code is not to be used to describe feelings about the services received (see **PPP Clinic Complaints** or **Quality of Services**).

PPP Service Delivery
General thoughts about or perspectives on PPP services and the integrated strategy of ART and PrEP delivery. This includes both positive and negative perspectives on the overall quality of services delivered at the PPP site, including whether services met couples' needs and expectations, and the impact this has (i.e., deciding to change clinical sites). In cases where there is overlap with **PPP Clinic Complaints**, choose only the most relevant code for the excerpt of text.

Previous Care
Descriptions of past experiences with health care at other facilities or clinics. These experiences, both positive and negative, may contribute to decisions to seek care elsewhere at the present time (i.e., the PPP site). Some examples include: spending all day at the clinic, aggressive treatment by health care staff, and inconvenient clinic hours or dispensing schedules. This code will often be paired with other codes (i.e., **Joining PPP**, **ART/ PrEP Uptake**).

ART/ PrEP Use Codes

Adherence Support
Encouragement by a partner, family member, friend and/or others to take one's pills. This can include reminders to take pills, assisting with medication collection/ pick-up. Support from health care workers should be coded **Counseling – Adherence**.

ART/ PrEP Use
Decisions, motivations and/or other reasons for choosing to initiate and use ART or PrEP. Reasons include the desire to: remain together, maintain one's health, prevent HIV in the relationship, etc.

Missed Doses
Descriptions of specific situations when pills were not taken. This may include intentional or unintentional reasons for missed doses, patterns for missing does and/or feelings about missed doses. Simple mention of a single missed dose will not be coded.

Modeling
Examples of couples taking ART and PrEP together at the same time, as a way of facilitating adherence and/or maintaining good relationships. This includes explanations that they must keep taking pills since their partner is also taking medication.

Partner Dosing
Perspectives on the study partner's adherence and/or dosing patterns. This may include descriptions of how the study partner does or does not take his/her pills.

PrEP Continuation
Explanations of and reasons for continuing to take PrEP longer than the typical or "prescribed" time (6 months following ART initiation in the partner). This may also include preferences for staying on PrEP, regardless of real or actual risk - i.e., the desire for additional protection, a lack of trust in the partner, concerns about fidelity or ART adherence, or because of individual risk outside of the serodiscordant

relationship (i.e., other partners of unknown HIV status). Specific concerns about stopping PrEP should be coded **PrEP Stop.**

PrEP Stop
Experiences of and perspectives on PrEP discontinuation, including concerns about stopping PrEP in the future and actual reasons for stopping PrEP – i.e., decreased risk, separation from partner, viral suppression in the partner, or because of clinical guidance from a health care worker.

Previous ART use
Experiences of taking ART in the past, including descriptions of how long ART was taken and reasons ART use was discontinued (if discontinued). Reasons for taking ART through PPP should be as **ART/ PrEP Use**.

Reluctance
Concerns and/or a hesitance to seek HIV treatment or prevention services, and/or to initiate or take ART or PrEP. This includes concerns about: being seen at an HIV clinic to collect PrEP (stigma), disclosing their status to partners, and ART/PrEP side effects.

HIV Prevention

Condom use
Reasons for using, or not using, condoms. This may include explanations about preferences for using condoms over PrEP, or using both PrEP and condoms together. PLANS TO USE

PrEP Attitudes
General opinions or perceptions about PrEP and its effectiveness as HIV prevention, including preferences for (or against) using PrEP over other prevention methods. This code should not overlap with the PrEP Use codes.

Risk perception
Attitudes or assessments about the chances of becoming HIV-infected, due to actual or perceived exposure to HIV, including thoughts, feelings or understandings of risk and the impact it has on one's decisions or behavior.

U=U
Understandings about and perspectives on the concept that viral suppression in the HIV-positive partner protects against HIV transmission in the negative partner. This includes confidence, trust, doubts and other positive or negative feelings.

Relationship Codes

Intimate Partner Violence (IPV)
Descriptions of harmful acts inflicted by the study partner, including sexual (i.e., forced sex or rape), physical (i.e., hitting, kicking, throwing things) or mental (i.e., verbal insults, or emotional manipulation) abuse.

Separation
Fears about or descriptions of separating from the study partner. This may include the reasons for and consequences of separation (i.e., going back to the village, loss of economic support, etc.)

Solidarity
Feelings of admiration, trust or respect for one's partner. This includes examples of encouragement, morale-boosting and other emotional support among couples in the face of serodiscordance, HIV or other life circumstances. Support for medication uptake or adherence should be coded **Adherence Support**. Examples of decisions to remain in the relationship should be coded **Stay Together**.

Stay Together
Examples of couples overcoming conflict related to serodiscordance or other issues (i.e., infidelity). This includes decisions to remain together and their renewed commitment towards improving their relationship.

Other Influence Codes

Access
Descriptions of the ways in which the ability to obtain HIV prevention and treatment services as scheduled, including ART and PrEP, are affected. This includes references to availability of medications, disruptions in transport and long distances which make timely refills impossible. When this overlaps with other codes, select the most specific code.

Alternative Refills
Alternative forms of medication collection when unable to pick up pills from the PPP site. This may include home delivery, community-based delivery sites or attending other clinics for ART or PrEP refills.

Change
Accounts of changes in experiences between interviews. This code will always be paired with other codes.

Food Insecurity
Descriptions of diminished access to desirable food and the impact it has on one's ability to take pills or seek health services, possibly as a direct result of the lockdown.

HIV Stigma
Fears of being (mis)identified as HIV-positive as a result of attending HIV clinics for medication refills.

Lockdown
Specific references to or examples of how the COVID-19 pandemic and ensuing lockdown impacted the daily lives of study participants. This code will most often be paired with other codes.

Mobility
Descriptions of the ability to move freely, including travel (i.e., being away from one's primary home) and transportation restrictions, and the impact on the couple's life. This may influence their ability to maintain their relationship or to access HIV prevention or treatment services.

Resource Scarcity
Descriptions of diminished financial resources or material goods and the impact on participants' lives (i.e., conflicts with partners or other family members), possibly as a direct result of the lockdown.
