# Supplementary material for: How PrEP delivery was integrated into public ART clinics in central Uganda: A qualitative analysis of implementation processes
Source: PLOS Glob Public Health. 2024 Mar 7;4(3):e0002916. doi: 10.1371/journal.pgph.0002916 (PMC10919847; doi:10.1371/journal.pgph.0002916)
Supplement: Attachment — Submitted filename: Wyatt_Response to Reviewers.pdf [file pgph.0002916.s009.pdf]

HARVARD MEDICAL SCHOOL

DEPARTMENT OF GLOBAL HEALTH AND SOCIAL MEDICINE

641 Huntington Avenue
Boston, Massachusetts 02115
Tel. 617-432-1707
FAX 617-432-2565

December 14, 2023

Sarah E. Brewer, PhD
Academic Editor
PLOS Global Public Health

Dear Dr. Brewer:

We thank you and your colleagues for your consideration of our manuscript entitled "How PrEP delivery was integrated into public ART clinics in central Uganda: A qualitative analysis of implementation processes" (PGPH-D-23-01404). We appreciate the thoughtful comments and suggestions from the reviewers included in your email, dated October 10, 2023. We are pleased to submit a revised version of the manuscript in response. We have addressed each concern in detail, with point-by-point responses *in italics*. Changes to the text are indicated through the use of **bold.**

Response to Reviewers' Comments

Reviewer #1: Thank you for the opportunity to review this important manuscript. This is a qualitative study near Kampala, Uganda. The study was nested within a stepped-wedged cluster randomized trial of the integration of PrEP and ART delivery for serodifferent couples from the perspectives (in-depth interviews) of 42/149 couples (83 interviews) and 36 healthcare providers from 12 health facilities. A second interview was conducted in a subset of 23 couples (45 interviews). Negative partners-initiated PrEP to support and preserve relationships, encouraging positive partners to adhere to ART.

Response: We appreciate the positive feedback.

(1) Introduction
a. Line 63 – "near elimination of HIV", not clear of the meaning, can be clarified by the authors to improve comprehension?

Response: We have clarified the meaning of this statement by revising the sentence. It now reads as follows:

> "Building on the results of the Partners PrEP Study, a clinical trial of PrEP efficacy [10], the Partners Demonstration Project implemented integrated delivery

> of ART and PrEP for serodifferent couples in Kenya and Uganda from 2012 to 2016. **Results showed** high levels of ART and PrEP uptake, **and virtually no incident HIV infection** [11,12]."

(2) Analyses and findings
a. Is there data on education and occupation available for serodifferent couples' members?

<u>Response</u>*: We thank the reviewer for raising this question. Information for describing participant characteristics was limited to what was collected as part of the behavioral research component. In the interest of reducing the burden on research participants, detailed sociodemographic characteristics, including information on education and occupation, were not included in data collection.*

b. Is there data on health professionals' cadres?

<u>Response</u>*: Our sample of 36 health care providers included 22 counselors, 3 clinicians, 8 nurses and 3 expert peers/ clients. We have revised the table and text to include information on professional roles for health care providers.*

c. Stigma is well described as one of the barriers to PrEP, any mention from the couples about fear of adverse effects?

<u>Response</u>*: Thank you for this comment. While the qualitative data with health care providers pointed to fear of stigma as a barrier to PrEP initiation (Lines 499-507), stigma did not emerge as an obstacle to PrEP use in the interviews with serodifferent couples. Fear of other adverse events – i.e., side effects from PrEP – also was not mentioned as a major barrier to PrEP use for HIV-negative members of couples in this qualitative sample. Those who experienced side effects noted they were manageable, and temporary.*

d. Was data saturation discussed/achieved?

<u>Response</u>*: We thank the reviewer for noting this omission. In response to this comment, we have added the following statement in bold to the text in Methods, in the section on Data Analysis (Lines 169-171):*

> "…The categories were analyzed inductively to identify their relationships to each other, and were grouped iteratively based on shared content. **All concepts relevant to program implementation that were mentioned by participants are represented in the categories; data saturation was achieved."**

Reviewer #2: Thank you for inviting me to review this manuscript. The article meets the main criteria for publication as articulated by the journal, in that, for example, it describes original research, is clearly written in English, and the study went through ethical review / an IRB etc. The qualitative sample is a robust size and is derived from a well-designed

stepped wedge cluster RCT and the description of the analyses are appropriate and detailed. I enjoyed reading the discussion, and feel that the conclusions are supported by the data. That said, there are a few main issues I would like to raise:

<u>Response</u>: *We appreciate the reviewer's overall enthusiasm for our manuscript. We address each concern in our responses, noted below.*

(1) This is a purposively selected sample and as such the data are not necessarily representative of the larger community of serodifferent couples (which the authors acknowledge). However, I would still like to know more about how the participants were selected (as well as a sense of response rates to the invitations to participate).

<u>Response</u>: *We appreciate this comment from the reviewer. The qualitative sample was drawn from a subset of 149 serodifferent couples who took part in the behavioral research component of the stepped wedge cluster randomized trial (N=1,381). The behavioral research component consisted of questionnaires completed at enrollment, three months and every six months thereafter, for a period of up-to-2 years. Questions on HIV prevention preferences, facilitators and barriers to ART or PrEP use, perception of HIV risk, sexual behavior, relationship power, alcohol use, internalized stigma, and modeling of PrEP use were assessed.*

Participants in the behavioral research component were a convenience sample of the subset of trial participants who initiated PrEP and ART (N=527). Facility-based staff identified couples as part of clinic visits during which PrEP and ART were offered. Couples were later approached by a member of the Partners PrEP Program study team to determine their willingness to participate in the behavioral research component. Individuals who agreed to take part in this research consented at the same time to future qualitative interviews. A total of 149 couples was enrolled in the behavioral component. Refusals to participate were not tracked. Quantitative data collected as part of the behavioral research component are being written up separately.

The qualitative sample was purposefully selected from participants in the behavioral research component with the following goals in mind. First, we sought to include in the qualitative sample up-to-50 couples from all clinical sites participating in the larger trial. Second, we worked to ensure the proportion of qualitative participants from each site reflected the proportion of participants in the behavioral research component from that site. Finally, we ensured the proportion of men and women included in the qualitative sample reflected the overall gender distribution of participants in the trial.

In response to the reviewer's suggestion, we have included the following bolded text to give more information about how the qualitative sample was selected and to clarify the refusal rate among couples invited to participate. We have also added Figure 1 to clarify how the qualitative sample fits into the larger participant populations (see Figure 1).

> "A subset ~~of 42 serodifferent couples~~ of the 149 couples participating in the behavioral research were purposefully selected to take part in qualitative

> interviews **(see Figure 1)**. The sampling scheme ~~had two goals~~ **sought to**: 1) include **up-to-50** couples from ~~each of~~ the 12 facilities **participating in the trial**; 2) **ensure the proportion of interview participants from each site reflected the proportion of behavioral research component participants from that site; and 3)** reflect the gender distribution of **participants in** the **trial** ~~overall research cohort~~. Members of each couple were contacted separately by ~~a research assistant (RA)~~ by phone to determine interest in participating. **Of the couples contacted, 8 refused to participate in a qualitative interview."**

(2a) The authors indicate that in fact many of the couples did not return for PrEP / continue on PrEP. This is a major challenge for the field in general, and should be highlighted more in the reporting of results and implications related to the current findings. If issues related to maintenance of relationships are key for PrEP initiation (as is reported here), would they not be for PrEP continuation / persistence? Why would this be the case?

<u>Response</u>: *We recognize and appreciate the importance of questions on PrEP continuation and discontinuation raised by the reviewer. Retention on PrEP is a major challenge to the field and we agree with the reviewer's suggestion that it would be useful to examine how couples' relationships affect PrEP continuation. However, this is beyond the scope of our present analysis.*

We have published extensively on PrEP use, adherence and discontinuation in our previous qualitative research on HIV serodifferent couples in Uganda [1-4]. In contrast, this analysis specifically addresses intervention delivery. Using two sources of qualitative data – interviews with health care providers and interviews with serodifferent couples – we unpack the process of integrating PrEP into existing ART services in public health facilities. Our emphasis in this manuscript is on offering insights to inform future program implementation, rather than addressing questions related to PrEP use.

Although processes of intervention delivery are the focus of this paper, we agree that the status of partnered relationships is likely an important driver of PrEP continuation and discontinuation, as the reviewer suggests. Some evidence of this appears in the manuscript. For example, ending a relationship or experiencing a temporary separation from one's partner (i.e., when traveling away from home for an extended period) were cited by some participants as reasons for discontinuing PrEP (see Lines 510-515). Another reason for stopping PrEP is the loss of intimacy, which we have explored in our previous work.

(2b) Further, the authors state at one point that providers reported that a lot of people resisted initiating (and not just remaining on) PrEP. How should that information be interpreted / what are the implications for the other PrEP initiation findings highlighted by the authors?

<u>Response</u>: *It may be helpful to point out that the large majority of individuals offered PrEP through the integrated delivery strategy initiated PrEP (527 out of 651, or 81%)*

[5]. That is, only 19% of HIV-negative partners in the overall trial declined PrEP. In order to be more accurate, we have changed the language in the text to read: "providers openly acknowledged the reluctance of **some** *HIV-negative partners to initiate PrEP," rather than "many."*

Providers in the qualitative study pointed to some possible interpretations for declining PrEP. These included: a) perception of low HIV risk on the part of prospective PrEP users, b) fear of being misidentified as living with HIV when taking PrEP, and c) concerns about pill burden (Lines 493-501). We discuss refusal to initiate PrEP as a challenge faced by providers in implementing the PrEP program. We are unable to report on PrEP decline from the perspectives of couples because all HIV-negative partners (N=41) in the qualitative interview sample initiated PrEP.

(3) This manuscript seems overly long to me, and I feel like the main findings that bring nuance/ a new perspective (and hence the main contributions) are getting somewhat lost. Many of the points / themes raised seem pretty generic (or known) to me – for example, the importance of high quality training or teamwork, or tailored counseling messages. The finding that is most interesting to me relates to how participants often perceived that initiating PrEP could / would preserve their relationships and that many partners with HIV recommitted to ART because the partner not living with HIV was willing to stay in the relationship and use PrEP. I would consider shortening the manuscript substantially, to highlight the important results.

<u>Response</u>*: We are glad to hear the reviewer finds our observations on relationship dynamics and PrEP use interesting and important. As pointed out above, we have published reports of these dynamics a number of times before [1-4]. For this and other reasons, we felt the most constructive direction to take in this analysis was to use our data to show how health care providers and serodifferent couples experienced integrated ART and PrEP delivery in public health clinics, in order to understand key implementation processes. The rationale for this is that an understanding of implementation processes can help to facilitate integration of new programs or delivery strategies into routine practice settings.*

This study is one of the first we know of to evaluate implementation processes for integrated ART and PrEP delivery. We believe that one of the main contributions of this manuscript is that it adds a new framing of couples' responses to implementers' efforts to make the integrated strategy appealing (or acceptable) to them, while also highlighting the implementer perspective. In order to make the focus on implementation more explicit, we have revised the description of data analysis to first present how the health care provider data were analyzed (i.e., before describing analysis of the couples' interview data) (Lines 153-161). We also revised the following two sentences in the Methods section:

> "The study design also included a qualitative component whose goals ~~were to characterize ART and PrEP use and~~ **include** examin**ing** the integrated delivery

approach, from the perspective of serodifferent couples and implementing health care providers."

"The **primary** goal of this analysis was to show how ~~study participants and~~ health care providers ~~experienced ART and~~ integrated PrEP delivery in**to** ~~public health clinics in central Uganda~~ **ART services**, in order to understand key implementation processes. **We also explored serodifferent couples' responses to the integrated strategy.**"

In response to the reviewer's recommendation to shorten the manuscript, we have done a careful edit, shortening it by more than 700 words. This included tightening the language, and deleting the paragraph on the impact of the pandemic on access to HIV prevention and treatment in the Discussion. Although we were unable to cut the suggested paragraphs in Results (the importance of high-quality training, teamwork and tailored counseling messages), we have tried to address the reviewer's concern while also preserving the integrity of our analysis. We believe that removing these suggested sections of text would undermine our explanation of implementation processes – specifically what made it possible (or feasible) for providers to integrate PrEP into existing ART services, from their point of view.

(4) Data are not really available to the public – they need to be requested and will 'be considered on a case by case basis.' While this is not uncommon for qualitative research, it is important to be aware that this is the case, given related requirements of the journal.

<u>Response</u>: *We thank the reviewer for requesting clarification about how the qualitative data are made available to the public. After carefully considering PLOS's Data Availability Policy, we have opted to provide access to our qualitative dataset upon request. The health care provider interviews, from which a large part of this analysis is drawn, include specific details about health care facilities (i.e., names, locations) and the roles of health care providers (i.e., job titles, cadres). If made public, this information could inadvertently reveal the identities of individual interview participants. In interviews with serodifferent couples, data that are not directly identifying may become identifying, when combined with information about health care facilities and the circumstances under which integrated ART and PrEP services were provided. Requests to access the data will be considered on a case-by-case basis so that we can maintain confidentiality of our research data and ensure we do not compromise participants' privacy. We appreciate the importance of data sharing for transparency and replicability of research findings, and will make every effort to honor such requests to access the data.*

(5) The following additional comments are categorized by manuscript section.

a) Abstract: My sense is that the abstract doesn't adequately highlight the most interesting / unique results. In addition, the results concerning relationship dynamics are not really explained clearly.

Response: We appreciate the reviewer's concern, and understand that it is consistent with the points made in 2a and 2b above. The abstract highlights the main points that emerged from the data analysis and is intended to address the manuscript's primary focus on intervention delivery and implementation processes. Relationship dynamics are presented in the context of couples' responses to health care providers' efforts to promote the integrated ART and PrEP delivery strategy.

b) Introduction: The text states that the intervention was provided in public health facilities under 'real world conditions.' While the intervention was implemented in public facilities it seems that the activities were implemented by specially trained staff supported over time by the PrEP study team. If that is correct, then one could argue that this is not really 'real world' conditions – please do clarify / nuance the related text.

Response: We thank the reviewer for this comment. As part of the launch of the PrEP program, health care personnel responsible for HIV treatment and prevention (counseling, laboratory functions or medication provision) at the public health facilities took part in a 2-day training adapted from the Uganda national PrEP training curriculum. Each facility participating in the program developed their own systems and methods for integrating PrEP into ART services. Once PrEP was introduced at a site, trained facility staff offered PrEP to eligible clients during routine service provision. In this way, the intervention attempted to approximate normal ("real-world") conditions within public health facilities. However, to avoid any confusion, we have removed the reference to "under real-world conditions" from the manuscript, as suggested by the reviewer.

c) Methods: The text states that transcripts were reviewed for quality. Can the authors clarify who reviewed them, how many people reviewed the transcripts, and how they were reviewed?

Response: Quality checks were performed by author MAW and/or EEP. Transcripts were assessed for quality in the following domains: 1) Interview technique: Were open-ended questions used? Did the interviewer probe to clarify meaning? 2) Interview content: Were the interview topics covered sufficiently, and in detail? and 3) Transcription accuracy: Were there grammatical errors? Is the meaning of the text clear in English?

We have clarified in the Methods that feedback was given specifically on data quality, and have added the initials of the authors who reviewed the transcripts, as indicated in bold below (Lines 145-149):

> "Interview transcripts were reviewed for quality **by author MAW or EEP**. Feedback on **data quality, including** interview technique, content and transcription accuracy was given **in** weekly teleconference meetings, over email and during in-person supervision visits by senior members of the research team (MAW, NCW, EEP)."

d) It would be useful to have some sort of conceptual framework, a guide for the types of

issues that were explored (in the context of this Implementation Science study / process evaluation.) Further, the text refers to a 'framework method' to 'visually display coded data' – could the authors explain this a bit?

<u>Response</u>: *The framework approach is an additional method for systematically reducing qualitative data [6]. It involves creating a spreadsheet, or matrix, in which cases (interviews) appear as rows, and key interview topics appear as columns. Within each "cell," data are summarized, including relevant quotes. This process facilitates analysis across a large number of interviews and topics as it enables the analyst to visually compare and contrast data within individual interviewees and across cases. In light of the reviewer's question about the use of conceptual frameworks, we can see how the reference to the "framework method" in the section on data analysis could be unclear. We have revised the text to now read:*

> "We also used a framework ~~method~~ **approach** [19] to **reduce** ~~visually display coded~~ the couples data. **This process involved visually displaying summaries of coded data** by **interview and** pre-designated interview topic in an analytic matrix. ~~The matrix enabled us to identify similarities and differences across interviews with serodifferent partners over time.~~"

We did not use a conceptual framework to guide the issues that were explored in this manuscript because the study design was intentionally inductive. Inductive study designs are widely accepted as alternatives to the more deductive, or "top down," approaches stemming from the use of a conceptual framework. An inductive approach to data collection and analysis does not rely on predesignated categories, thus allowing novel insights and discoveries to emerge from the data. One new insight that emerged from our analysis, for example, is the significance of the "meaning" of PrEP as a component of intervention acceptability for couples.

We agree with the reviewer that conceptual frameworks can be useful in contextualizing research findings. In the Discussion, we considered several frameworks for understanding acceptability, including the Theoretical Framework of Acceptability (Lines 572-583). We also discussed our findings in relation to Proctor and colleagues' "outcomes" framework for evaluating implementation processes (Lines 585-596). Our work highlighted the multidimensionality of two of these outcomes – acceptability and feasibility – suggesting these implementation concepts are interrelated in complex ways.

e) Results: Given that only 10% of initiators continued PrEP at 6 months, the text should very much highlight that these results are only about PrEP initiation and not PrEP 'use' more broadly or continuation.

<u>Response</u>: *We thank the reviewer for this comment. This outcome refers to the proportion of PrEP initiators in the overall stepped wedge cluster randomized trial of serodifferent couples who continued PrEP at 6 months [5]. We included this information in the Methods as part of the study background and design to provide more context for the qualitative research (Lines 97/98). High rates of discontinuation of PrEP were expected*

in the overall trial. The qualitative results presented here are not intended to explain the trial results; rather they are meant to paint a picture of the implementation processes which enabled public health facilities to successfully integrate PrEP delivery into their existing HIV prevention and treatment programs. As noted above, we have now included Figure 1 to show how the qualitative study is nested within the research that was conducted in the trial.

f) Tables 1 and 2 aren't so useful, to my mind, as they are reporting basic percentages that are also described in the text.

<u>Response</u>: *We thank the reviewer for this observation. In response, we have reorganized the tables, opting to present only gender, age and marital status for couples, and including this information in a revised Table 1, along with the sociodemographic data for health care providers.*

g) Regarding the theme of providers being concerned about additional work / burden, this is a real and commonly reported finding, but I am not quite clear how it is it relevant for the main question on how to integrate PrEP into ART programming. Can the authors clarify?

<u>Response</u>: *Increased workloads and insufficient staffing were important concerns for providers tasked with PrEP provision at participating public health facilities. Providers felt that the addition of new responsibilities made it more challenging to implement the integrated delivery program at the facility-level (see Lines 296-299). Our data do not allow us to explore how the concerns reported here impact the process of integrating PrEP into ART services, however, it would be worthwhile addressing this question in future research.*

h) Discussion: The summary in the beginning of the discussion is quite generic regarding provider feedback – the contribution of these findings is not quite clear to me.

<u>Response</u>: *The summary paragraph in the Discussion was intended to highlight the key findings of our analysis. In our earlier responses to reviewer comments, we have sought to clarify that the emphasis of this manuscript is on identifying and characterizing key implementation processes. The main contribution of the health care provider data is they deepened our understanding of processes that explain how PrEP delivery was integrated into existing ART services. For example, providers' appreciation of the meaning of relationships for serodifferent couples contributed to tailored counseling messages that promoted interest in the integrated delivery strategy. Couples, in turn, responded by initiating PrEP and recommitting to ART adherence.*

This completes our responses. Citations for materials referenced in our responses appear below.

Again, we thank the reviewers for their helpful comments and suggestions. We believe the manuscript to be significantly strengthened as a result of the revision process, and we hope you will now find it suitable for publication.

Sincerely,

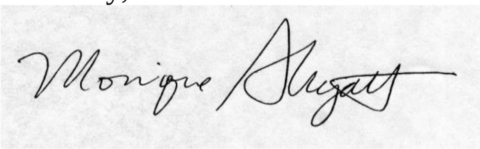

Monique A. Wyatt, BA
Department of Global Health and Social Medicine
Harvard Medical School

References Cited:

1. Ware NC, Wyatt MA, Haberer JE, Baeten JM, Kintu A, Psaros C, et al. What's love got to do with it? Explaining adherence to oral antiretroviral pre-exposure prophylaxis for HIV-serodiscordant couples. J Acquir Immune Defic Syndr. 2012;59(5):4630468. doi: 10.1097/QAI.0b013e31824a060b

2. Ware NC, Pisarski EE, Nakku-Joloba E, Wyatt MA, Muwonge TR, Turyameeba B, et al. Integrated delivery of antiretroviral treatment and pre-exposure prophylaxis to HIV-1 serodiscordant couples in East Africa: a qualitative evaluation study in Uganda. J Int AIDS Soc. 2018;21(5):e25113. doi: 10.1002/jia2.25113

3. Nakku-Joloba E, Pisarski EE, Wyatt MA, Muwonge TR, Asiimwe S, Celum CL, et al. Beyond HIV prevention: everyday life priorities and demand for PrEP among Ugandan HIV serodiscordant couples. J Int AIDS Soc. 22:e25225. doi: 10.1002/jia2.25225

4. Gilbert HN, Wyatt MA, Pisarski EE, Muwonge TR, Heffron R, Katabira ET, et al. PrEP Discontinuation and Prevention-Effective Adherence: Experiences of PrEP Users in Ugandan HIV Serodiscordant Couples. J Acquir Immune Defic Syndr. 2019 November 01; 82(3): 265–274. doi:10.1097/QAI. 0000000000002139

5. Heffron R, Muwonge TR, Thomas KK, Nambi F, Nakabugo L, Kibuuka J, et al. PrEP uptake and HIV viral suppression when PrEP is integrated into Ugandan ART clinics for HIV-negative members of HIV-serodifferent couples: a stepped wedge cluster randomized trial. EClinicalMedicine. 2022;52:101611. doi: 10.1016/j.eclinm.2022.101611

6. Gale NK, Heath G, Cameron E, Rashid S, Redwood S. Using the framework method for the analysis of qualitative data in multi-disciplinary health research. BMC Med Res Methodol. 2013;13: 117–8. doi: 10.1186/1471-2288-13-117
